# Adaptive collaborative feature fusion and shape-aware optimization for multi-scale chest lesion detection

**DOI:** 10.1038/s41598-026-56315-w

**Published:** 2026-06-12

**Authors:** Yubo Yuan, Chengkang Liu, Qingwen Feng, Li Zhang, Kun Yang, Shengyue Cheng

**Affiliations:** 1 School of Information Engineering, Zhengzhou University of Science and Technology, Zhengzhou, 450064 China; 2 Engineering Technology Research Center for Visual Big Data Processing and Control, Zhengzhou University of Science and Technology, Zhengzhou, 450064 China; 3 Key Laboratory of Visual Big Data Processing and Application, Zhengzhou University of Science and Technology, Zhengzhou, 450064 China

**Keywords:** Computational biology and bioinformatics, Diseases, Engineering, Health care, Mathematics and computing, Medical research

## Abstract

Chest diseases remain a major cause of global morbidity and mortality, and accurate detection from chest X-ray images is critical for early diagnosis and clinical decision-making. However, large variations in lesion scale, morphology, and spatial distribution pose significant challenges for automated detection systems, particularly in identifying small lesions and achieving precise localization. To address these issues, we propose a multi-scale chest lesion detection method based on adaptive collaborative feature fusion and shape-aware optimization. The method enhances multi-scale feature modeling and introduces shape structural constraints to improve detection accuracy and localization robustness in complex anatomical environments. Specifically, a Context-Embedded Feature Enhancement Network is designed to jointly capture global anatomical context and local lesion characteristics, strengthening lesion representation. An Adaptive Feature Focusing Network further improves multi-scale feature representation through adaptive spatial feature aggregation, enabling more effective detection of small lesions. In addition, a shape-aware optimization strategy integrating normalized Wasserstein distance with shape-weighted constraints improves localization stability and bounding box regression accuracy for irregular lesions. Compared with state-of-the-art methods, the proposed method achieves improvements of 6.3% in *mAP*, 6.4% in small-lesion *mAP*, and 8.6% in mean recall on VinDr-CXR, as well as improvements of 4.2% in *mAP* and 7.8% in mean recall on ChestX-ray8, demonstrating its effectiveness and generalization capability for multi-scale chest lesion detection.

## Introduction

Chest diseases have become one of the leading causes of morbidity and mortality worldwide, posing a significant challenge to global public health. According to statistics reported by the World Health Organization (WHO), thoracic diseases such as lung cancer, pneumonia, and tuberculosis account for a substantial proportion of annual deaths, underscoring the urgent need for an accurate and timely diagnosis^1^. Early detection of thoracic diseases is critical for improving patient survival rates, treatment effectiveness, and long-term prognosis.

Medical imaging has become the cornerstone of early diagnosis of thoracic disease, with chest X-ray (CXR) and computed tomography (CT) as the most widely used modalities in clinical practice. These imaging techniques provide detailed structural information about the thoracic cavity, enabling clinicians to analyze lesion characteristics, including morphology, size, density, and spatial distribution. Beyond common thoracic diseases, including lung cancer, pneumonia, and tuberculosis, chest imaging also supports the diagnosis of various other conditions, such as pneumothorax, emphysema, and cardiovascular abnormalities^2^.

Although chest imaging holds significant value in clinical diagnosis, traditional image analysis relies heavily on manual interpretation by radiologists. Manual interpretation is inevitably constrained by individual experience, workload, and fatigue factors. Thoracic diseases often present in early stages as minute, low-contrast, or nonspecific abnormalities that can easily be confused with normal anatomical structures. Particularly in high-intensity clinical settings, manual diagnosis is prone to missed or misdiagnosed cases. These limitations have driven researchers to continuously explore automated and intelligent methods for detecting thoracic diseases^3^.

With the rapid advancement of deep learning, especially convolutional neural networks (CNNs), automated analysis of medical images has achieved remarkable progress. CNN-based models have demonstrated strong capability in hierarchical feature extraction, enabling effective representation learning across different spatial scales^4^. Numerous studies have leveraged classical architectures such as ResNet and DenseNet to achieve promising performance in thoracic disease classification and detection^5^. However, despite these achievements, several critical challenges remain unresolved.

First, lesions in chest images exhibit substantial variability in size, shape, contrast, and location. Small lesions and low-contrast abnormalities are difficult to detect because their visual features are easily overwhelmed by complex anatomical backgrounds^6^. Second, existing detection frameworks often suffer from inadequate multi-scale feature fusion and an imbalance between global semantic information and local fine-grained details. This limitation leads to reduced sensitivity to small lesions and unstable performance under complex anatomical conditions^7^. Third, most current bounding box regression methods rely on IoU-based metrics that primarily measure spatial overlap and fail to model geometric shape variations explicitly. As a result, localization accuracy for irregularly shaped lesions remains suboptimal^8^.

In recent years, Vision Transformers (ViTs) have been introduced into medical image analysis, offering a new paradigm for global context modeling through self-attention mechanisms. Transformer-based and hybrid CNN-Transformer architectures have demonstrated improved performance in various thoracic disease detection tasks by capturing long-range dependencies and global anatomical relationships. Nevertheless, pure Transformer architectures may overlook fine-grained local details, while hybrid models often introduce increased computational complexity^9^. Consequently, achieving an effective balance between global context modeling and preservation of local detail remains a key research challenge.

Another fundamental issue in thoracic lesion detection lies in scale diversity. Lesions in chest images range from minute nodules to large consolidations, making single-receptive-field feature extraction insufficient^10^. Feature Pyramid Networks (FPNs) and their variants have been widely adopted to address multi-scale detection by fusing deep semantic features with shallow spatial details. However, traditional FPNs employ static fusion strategies with fixed weights, which fail to adapt to heterogeneous feature distributions of lesions on different scales. This limitation often leads to semantic misalignment and characteristic degradation, particularly in small lesions.

Moreover, contextual information is crucial for thoracic image interpretation. Radiologists do not rely solely on local lesion appearance but also consider anatomical context, such as spatial relationships between lesions and surrounding tissues, bilateral symmetry of lung fields, and relative anatomical positions. While early CNN-based approaches attempted to model context by increasing receptive fields through deeper architectures, they remain inefficient for capturing long-range dependencies^11^. Although self-attention and non-local mechanisms have improved global context modeling, effectively integrating contextual awareness with local feature fidelity remains challenging.

Finally, accurate lesion localization is essential for clinical applicability. Conventional IoU-based loss functions suffer from gradient instability when predicted and ground-truth boxes do not overlap, which is common for small or irregular lesions. More importantly, these metrics ignore shape similarity and boundary structure, limiting their effectiveness in modeling the geometric complexity of thoracic lesions. Addressing these issues requires a localization strategy that is both scale-invariant and shape-aware.

To overcome the aforementioned challenges, this paper proposes an adaptive collaborative feature fusion and shape-aware optimization framework for thoracic disease detection. The main contributions of this work can be summarized as follows:

- We propose a Context-Embedded Feature Enhancement Network (CEFEN) to collaboratively model global anatomical context and local fine-grained details, enabling robust lesion representation under complex thoracic backgrounds.
- We introduce an Adaptive Feature Focusing Network (AFFN) to dynamically aggregate multi-scale features through adaptive spatial fusion, significantly enhancing detection sensitivity to small lesions.
- We develop a Shape-Aware Optimization Strategy (SAOS) that integrates the normalized Wasserstein distance with medical shape-weighted constraints, improving localization robustness and accuracy for irregularly shaped lesions.

Extensive experiments on the public VinDr-CXR and ChestX-ray8 datasets demonstrate that the proposed method consistently outperforms representative approaches in terms of detection accuracy, small-lesion sensitivity, and localization precision, while maintaining an efficient model complexity.

## Related work

Recent advances in thoracic disease detection have primarily focused on three closely related research directions: feature representation learning using convolutional neural networks and attention mechanisms, global contextual modeling with Transformer-based or hybrid architectures, and lesion localization via object detection frameworks with multi-scale feature fusion. These studies have significantly improved detection accuracy and robustness; however, limitations remain in balancing global anatomical context with local fine-grained details, adapting to extreme lesion scale variations, and achieving stable localization for irregularly shaped lesions. Accordingly, this section reviews representative studies from the three perspectives outlined above, summarizes the main research progress in this field, and discusses the remaining unresolved issues.

### CNN-based and attention-enhanced methods for chest disease detection

Convolutional neural networks (CNNs) have been widely adopted as the foundational framework for thoracic disease detection from chest X-ray (CXR) images. Early studies primarily relied on classical architectures, such as ResNet and DenseNet, for end-to-end feature extraction and disease classification. Balmuri et al.^12^ proposed an ensemble transfer learning framework for automated multi-disease detection, incorporating optimization strategies to reduce misclassification in complex clinical scenarios. Ahad et al.^13^ further introduced a fusion model combining an enhanced DenseNet201 with a custom CNN, achieving improved accuracy and interpretability through Grad-CAM visualization.

To enhance representational capacity, attention mechanisms have been increasingly integrated into CNN architectures. Habeeb et al.^14^ developed a diagnosis-optimized ResNet-50 for lung cancer detection from CXR images. Akshat et al.^15^ proposed ResAttn-Net, incorporating Squeeze-and-Excitation blocks and multi-scale feature fusion. Goncalves et al.^16^ introduced DualAttentionNet, combining DenseNet and ResNet backbones with attention modules.

Despite their effectiveness, most CNN-based approaches rely heavily on global feature representations and exhibit limited sensitivity to subtle local patterns. As reported in^17^, these models are prone to false positives and missed detections when dealing with small or low-contrast lesions in complex anatomical backgrounds.

### Transformer and hybrid architectures with global context modeling

The introduction of Vision Transformers (ViTs) has provided a new paradigm for thoracic image analysis by enabling long-range dependency modeling through self-attention mechanisms. Singh et al.^18^ proposed a ViT-based framework for pneumonia detection in CXR images. Mezina et al.^19^ introduced an Inception-ViT hybrid model for detecting long-term COVID-19 conditions.

To compensate for the limited local modeling capability of pure Transformers, hybrid CNN-Transformer architectures have been explored. Bhuvanya et al.^20^ proposed the X-Vision model, integrating ViT-based global context modeling with XceptionNet’s fine-grained local feature extraction. Aljaddouh et al.^21^ further demonstrated the effectiveness of Transformer-based models in medical signal analysis.

Although Transformer-based and hybrid architectures often outperform traditional CNNs^22^, pure Transformer models may overlook fine-grained local details, while hybrid models usually introduce higher computational complexity. Balancing global context modeling and local detail preservation remains a challenging issue.

### Object detection, multi-scale feature fusion, and shape-aware localization

Object detection frameworks, particularly YOLO-based models, have been widely employed for thoracic lesion localization. Hao et al.^23^ proposed YOLO-CXR to improve the localization of small lesions. Zeng et al.^24^ introduced YOLO-ED for lung cancer detection in CT images. Subsequent studies further enhanced detection accuracy and efficiency through attention mechanisms and architectural optimization^25,26^.

To address scale variation, feature pyramid networks (FPNs) have been widely adopted^27,28^. However, static fusion strategies often lead to semantic misalignment and degraded representation of small lesions. Moreover, conventional IoU-based loss functions focus solely on spatial overlap and fail to capture shape variability, leading to unstable localization for irregular lesions. Multi-scale modeling has also been widely investigated beyond medical object detection, demonstrating its general effectiveness across different vision tasks. For example, CGD-TraP^29^ introduces a condition-guided diffusion framework for multi-modal pedestrian trajectory prediction, showing the importance of incorporating prior conditions for complex visual prediction tasks. MB-TaylorFormer v2^30^ adopts a multi-branch linear Transformer with multi-scale patch embedding to capture coarse-to-fine image features efficiently in image restoration. ESTINet^31^ explores enhanced spatio-temporal interaction learning for video deraining, while DDMSNet^32^ exploits deep dense multi-scale representations with semantic and depth priors for snow removal. DBLRNet^33^ further demonstrates the effectiveness of jointly modeling spatial and temporal information in video deblurring. These studies indicate that multi-scale or hierarchical representation learning is a general and effective strategy for handling complex visual variations.

However, chest lesion detection differs from these general vision tasks because lesions in chest radiographs often exhibit large scale variations, weak visual contrast, ambiguous boundaries, and irregular morphologies. Therefore, directly applying generic multi-scale fusion strategies may be insufficient for robust lesion detection. In this study, the proposed AFFN is designed to adaptively aggregate spatial features across different lesion scales, while CEFEN enhances global anatomical context and local lesion details. In addition, SAOS introduces shape-aware localization constraints to improve bounding-box regression for irregular lesions. In this way, the proposed framework extends the idea of multi-scale modeling to the specific challenges of chest lesion detection.

## Methods

**Figure 1:**
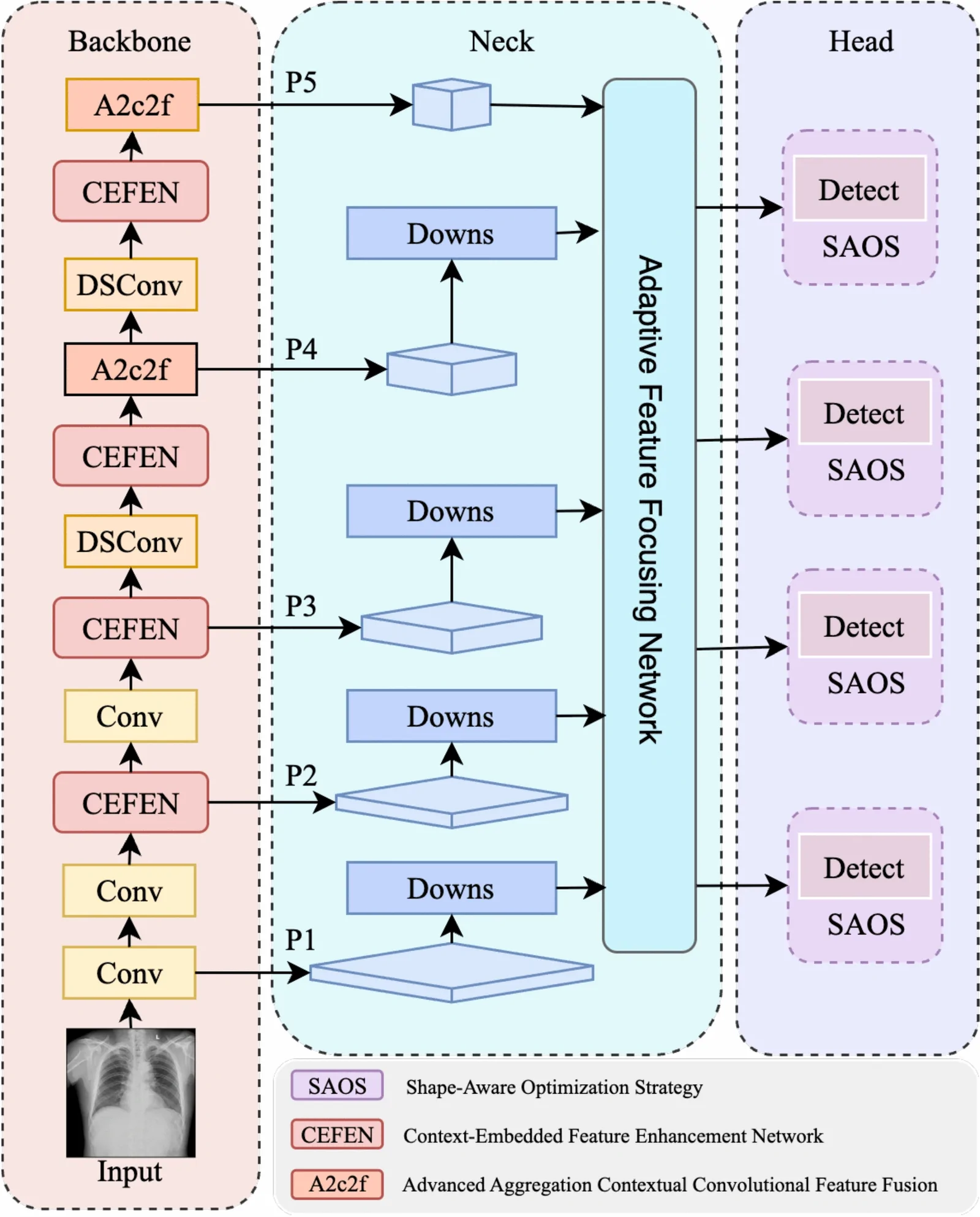
Overall architecture of the proposed framework. CEFEN is integrated into the backbone to enhance contextual and local lesion features, AFFN serves as the neck for adaptive multi-scale feature fusion, and SAOS is used as the bounding-box regression loss during training. The detection head outputs lesion categories and bounding boxes.

Due to the high variability in lesion size, morphology, and location in chest images, particularly the frequent occurrence of missed detections and inaccurate localization of minute lesions, an adaptive collaborative feature fusion and shape-aware optimization detection method is proposed. The overall framework is illustrated in Fig. 1. The proposed detector follows a standard backbone-neck-head architecture, and the three proposed modules are integrated into different stages of the baseline detector. Specifically, the Context-Embedded Feature Enhancement Network is embedded into the backbone to capture global contextual information while preserving critical local details, thereby improving the representation of small lesions in complex anatomical backgrounds. The Adaptive Feature Focusing Network is used as the feature fusion neck. By refining the asymptotic feature pyramid network architecture, AFFN introduces adaptive spatial fusion operations to enhance lesion feature aggregation across different scales. Finally, the Shape-Aware Optimization Strategy is applied in the detection head as the bounding-box regression objective. By combining normalized Wasserstein distance and shape-weighted constraints, SAOS improves the localization accuracy of minute and irregular lesions.

### Context-embedded feature enhancement network

**Figure 2:**
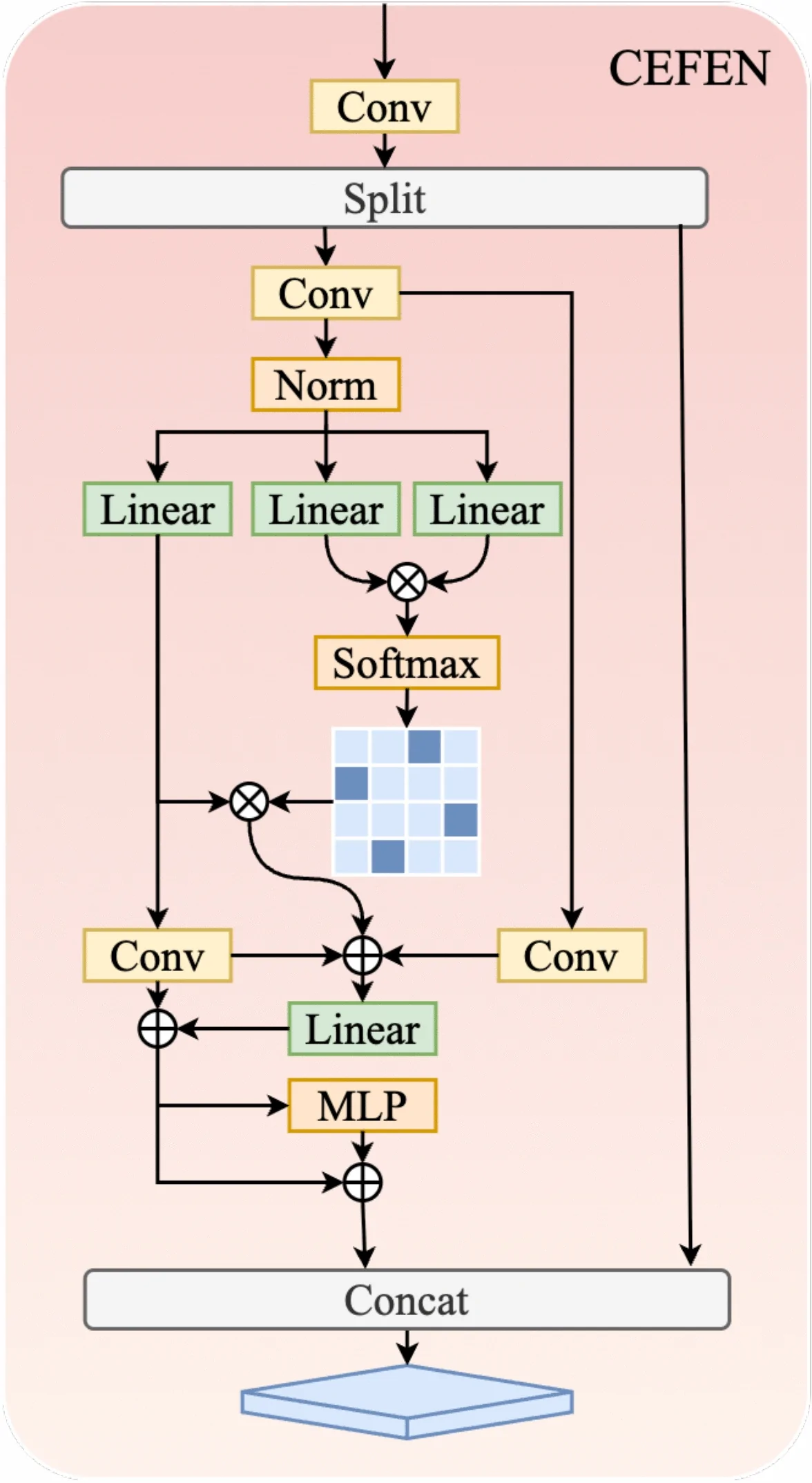
Network architecture diagram for contextual embedding feature enhancement.

In chest imaging disease detection, it is challenging to capture global semantic information while preserving critical local details simultaneously. Most existing detection methods rely on deep features to obtain global contextual information. However, as network depth increases, shallow spatial details are prone to gradual loss during propagation, leading to insufficient accuracy in identifying and localizing minute lesions. Particularly within complex thoracic anatomical structures, the imbalanced fusion of local and global features limits the model’s ability to perceive small lesions and low-contrast abnormalities, ultimately leading to missed detections and false positives. To address this issue, this study proposes a Context-Embedded Feature Enhancement Network (CEFEN), as shown in Fig. 2, to efficiently capture global information and local details. For clarity and notation consistency, the main symbols used in the method section are unified as follows. Pi denotes the feature map extracted from the i-th backbone stage, $F_i$ denotes the fused feature map at the i-th feature fusion level, $B_p$ and $B_g$ denote the predicted and ground-truth bounding boxes, respectively. Unless otherwise specified, *i* represents the feature level index.

When extracting lesion features from medical images (backbone), the high pixel dimensions of raw chest images and their abundant redundant information (such as background noise and non-lesion tissue textures) make them unsuitable for direct use in subsequent refined feature analysis. Therefore, after resizing to a uniform dimension, the chest image is represented as $I \in \mathbb {R}^{3 \times H \times W}$, where H and W denote the height and width of the input image, respectively, and 3 denotes the number of RGB channels. Following processing by the basic convolutional layer, the initial feature map $P_1$ is obtained, as shown in Equation(1).

1 $$\begin{aligned} P_1 = f_{\text {conv-int}}(I) \end{aligned}$$

where $f_{\text {conv-int}}(\cdot )$denotes the input preprocessing operation, employing a *3× 3*convolution kernel with a stride of 2 to achieve dimension reduction; $P_1$ represents the initial feature map.

To achieve parallel optimization of global-local features, CEFEN was designed based on the dedicated path specialization concept, as shown in Fig. 2. For the initial feature map $P_1$, we first perform a feature transformation convolution. Then, via the Split operation, we decompose the feature map $X_1$ along the channel dimension into two sub-features, $X_{1a}$ and $X_{1b}$, thereby constructing a dual-branch structure-the attention-enhancement branch and the detail-preservation branch. The implementation process is shown in Equation (2).

2 $$\begin{aligned} \begin{aligned} X_1 = f_{\mathrm {conv\text {-}trans}}(P_1), \quad \\ (X_{1a}, X_{1b}) = \textrm{Split}(X_1) \end{aligned} \end{aligned}$$

where $f_{\text {conv-trans}}(\cdot )$ denotes the feature transformation convolution operation, employing a *1× 1* convolution kernel to reduce computational load with stride *s=1*; *Split(· )* represents the channel splitting operation, which is the inverse of Concat; $X_1$ denotes the transformed feature map, where $X_1 \in \mathbb {R}^{C_1 \times H_1 \times W_1}$; $X_{1a} \in \mathbb {R}^{C_{1a} \times H_1 \times W_1}$ represents the global-oriented subfeature ($C_{1a} = C_1/2$), fed into the attention-enhancing branch. This branch focuses on capturing global anatomical semantics, such as the spatial distribution of large-scale structures like lung lobes or the heart, thereby assisting in narrowing the lesion search range; $X_{1b} \in \mathbb {R}^{C_{1b} \times H_1 \times W_1}$ represents detail-oriented sub-features ($C_{1b} = C_1/2$), fed into the detail-preservation branch. This branch focuses on retaining local lesion details, such as the edge textures of micro-nodules and the gray-scale variations in low-contrast lesions.

To enhance the semantic precision of subsequent attention computations, the attention enhancement branch first performs intermediate feature extraction. It refines mid-level semantics through the local perception capability of *3× 3* convolution kernels, as shown in Equation (3). Simultaneously, Layer Normalization (LN) is introduced to address gradient explosion/vanishing issues caused by extreme fluctuations in feature mean and variance during training. LN stabilizes training by standardizing the feature distribution, as shown in Equation (4).

3 $$\begin{aligned} \begin{aligned} X_{2a}&= f_{\text {conv-mid}}(X_{1a}) \end{aligned} \end{aligned}$$

4 $$\begin{aligned} \begin{aligned} \hat{X}_{2a}&= \text {Norm}(X_{2a}) = \frac{X_{2a} - \mathbb {E}[X_{2a}]}{\sqrt{\text {Var}(X_{2a}) + \varepsilon }} \cdot \gamma + \beta \end{aligned} \end{aligned}$$

where $X_{2a}$ represents the intermediate feature map, with the same number of channels C, height H, and width W as $X_{1a}$; $f_{\text {conv-mid}}(\cdot )$ employs a *3× 3* convolution kernel with stride *s=1*; $\text {Norm}(\cdot )$: layer normalization operation function; $\mathbb {E}[X_{2a}]$ denotes the feature mean; $\text {Var}(X_{2a})$ represents the feature variance; *\varepsilon* is a small value preventing the denominator from becoming zero; *γ* and *β* are learnable scaling and offset parameters; $\hat{X}_{2a}$ is the normalized feature map, sharing the same number of channels, height H, and width W as $X_{2a}$, with a more stable distribution suitable for subsequent linear transformations.

Subsequently, to achieve key information filtering, features for Query, Key, and Value are generated through three linear layers. Attention weights are computed via dot product operation followed by Softmax normalization, as shown in Equation (5).

5 $$\begin{aligned} \begin{aligned} Q&= L_1(\hat{X}_{2a}) \\ K&= L_2(\hat{X}_{2a}) \\ V&= L_3(\hat{X}_{2a}) \end{aligned} \end{aligned}$$

Where $L_1(\cdot ),L_2(\cdot )$ and $L_3(\cdot )$ are independent linear transformation layers,each comprising weight matrix multiplication plus bias term addition; *Q* represents query features used to compute similarity with *K* for locating key regions; *K* denotes key features representing semantic attributes, whose similarity with *Q* determines attention weights; *V* serves as value features, acting as the target for attention weights and carrying the semantic information requiring enhancement.

The similarity between *Q* and *K* is computed via dot product operations to obtain the weight for each positional feature. Subsequently, Softmax normalization ensures that the sum of weights equals 1, enabling subsequent weighting operations to focus on semantically significant regions–such as areas within lung lobes where lesions may exist–while downweighting irrelevant regions, such as the thoracic cage and background. The computational process is illustrated in Equation (6).

6 $$\begin{aligned} A_{\text {atten}} = \text {Softmax}\left( \frac{K^T \cdot Q}{\sqrt{d_k}} \right) \end{aligned}$$

Here, $A_{\text {atten}}$ denotes the attention weight matrix, where each element $A_{\text {atten}}(i,j)$ represents the attention weight from position *j* to position *i*. $K^T$ is the transpose of the key feature *K*, swapping its channel and spatial dimensions to ensure computability with the dot product of *Q*. $d_k$ is the key feature dimension used to scale the dot product result to prevent numerical overflow. ${\sqrt{d_k}}$ serves as the scaling factor, mitigating the vanishing gradient issue in the Softmax function due to large dot products as $d_k$ increases.

Applying attention weights to the value feature *V*, as shown in Equation (7), enhances key regional features with high weights and suppresses irrelevant regional features with low weights through multiplication by the weight matrix $A_{\text {atten}}$. This achieves a focus on global contextual information.

7 $$\begin{aligned} X_{3a} = V \cdot A_{\text {atten}} \end{aligned}$$

Where $X_{3a}$ represents the attention-weighted feature map, semantically highlighting information from globally critical regions.

Attention-weighted $X_{3a}$ enhances global information but may weaken original local details in $X_{1a}$, such as the edges of minute lesions. Therefore, $X_{3a}$ undergoes convolutional refinement while $X_{1a}$ retains original details through direct convolution. Element-wise summation then fuses both to achieve a balance between global focus and local preservation. The computational process is illustrated in Equation (8).

8 $$\begin{aligned} \begin{aligned} X_{4a}&= f_{\text {conv-fuse1}}(X_{3a}) \\ \hat{X}_{4a}&= f_{\text {conv-fuse2}}(X_{1a}) \\ X_{5a}&= X_{4a} + \hat{X}_{4a} \end{aligned} \end{aligned}$$

Where $f_{\text {conv-fuse1}}(\cdot )$ and $f_{\text {conv-fuse2}}(\cdot )$ are both *3× 3* convolutional operations, used respectively for refining attention features and preserving original details; $X_{4a}$ denotes the refined attention feature map; $\hat{X}_{4a}$ denotes the feature map preserving original details; $X_{5a}$ denotes the fused feature map, simultaneously incorporating global attention information and original local details.

To further enrich feature expression, linear transformations and multilayer perceptron (MLP) transformations were applied to the fused feature $X_{5a}$. The results from both transformations were then combined to enhance nonlinear representation. This approach preserves the fundamental semantics of $X_{5a}$ while strengthening its ability to represent complex anatomical structures, such as overlapping lung lobes and lesions. The computational process is illustrated in Equation (9).

9 $$\begin{aligned} \begin{aligned} X_{6a}&= L_4(X_{5a}) \\ X_{7a}&= f_{\text {mlp}}(X_{6a}) \\ X_{8a}&= X_{6a} + X_{7a} \end{aligned} \end{aligned}$$

Where $L_4(\cdot )$ is an independent linear transformation function designed to preserve the fundamental semantics of $X_{5a}$ and prevent information distortion caused by excessive nonlinearity; $f_{\text {mlp}}(\cdot )$ is a multilayer perceptron operation function that introduces nonlinearity via the ReLU activation function to fit complex feature correlations, such as gray-level differences between lesions and surrounding tissues; $X_{6a}$ and $X_{7a}$ represent the feature maps after linear and MLP transformations, respectively; $X_{8a}$ is the final output feature map of the attention-enhanced branch, combining fundamental semantic information with complex nonlinear representations to provide precise global contextual information.

The attention enhancement branch preserves some details through fusion operations but remains primarily context-aware. The detail retention branch refines the local semantics of $X_{1b}$ via convolutional operations, such as enhancing the edges of minute nodules and the grayscale texture of low-contrast lesions. This prevents excessive transformations from causing detail loss, ensuring sufficient local information is available for subsequent reconstruction. As shown in Equation(10).

10 $$\begin{aligned} X_{2b} = f_{\text {conv-detail}}(X_{1b}) \end{aligned}$$

Where $f_{\text {conv-detail}}(\cdot )$ is a lightweight convolution operation employing a *1× 1* convolution kernel, reducing parameters and computational load with stride *S=1*; $X_{2b}$ represents the final output feature map of the detail-preserving branch, whose kernel retains critical local information such as the edges of minute nodules and patchy texture patterns.

Finally, by concatenating along the channel dimension, the attention-enhanced branch $X_{8a}$ is fused with the detail-preserving branch $X_{2b}$, as shown in Equation(11), achieving a collaborative representation of global semantics and local details.

11 $$\begin{aligned} P_2 = \text {Concat}(X_{8a}, X_{2b}) \end{aligned}$$

Where $P_2$ represents the final output feature map from the Context Embedded Feature Enhancement Network (CEFEN). This feature incorporates both global anatomical semantics, such as lung lobes, to narrow the lesion search scope, while preserving local details of minute lesions to enhance detection rates. It can be directly fed into the subsequent Adaptive Feature Focusing Network for scale feature fusion. $\text {Concat}(\cdot )$ denotes a channel-wise concatenation operation, ensuring the output features simultaneously incorporate both global and local information.

To obtain the intermediate-level transitional feature $P_3$, we further integrate semantic information from the deep modules of the backbone on top of $P_2$, achieving a smooth transition from local details to intermediate-level semantics. The computational process is shown in Equation(12).

12 $$\begin{aligned} P_3 = \text {CEFEN}\left( \text {Conv}(P_2)\right) \end{aligned}$$

Where $P_3$ represents the intermediate transitional features extracted further; ${CEFEN}(\cdot )$ denotes the Context Embedding Feature Enhancement Network model, whose computation process is shown in Equation (2) to Equation (11);${Conv}(\cdot )$ refers to a 1*×*1 convolution used to match the number of feature channels.

$P_3$ reduces computational complexity through depthwise separable convolutions (DSConv)^34^ while integrating more abstract global semantics. It emphasizes cross-scale feature correlations for larger lesions and generates the cross-scale semantic correlation layer $P_4$. This is achieved via the adaptive channel-to-feature fusion module A2c2f^35^, which dynamically adjusts channel fusion ratios based on lesion probabilities within feature regions. The computational process is illustrated in Equation (13).

13 $$\begin{aligned} \begin{aligned} X_{P_4\text {-}Down}&= \text {PointwiseConv}\left( \text {DSConv}\left( \text {MaxPool}(P_3) \right) \right) \\ P_4&= A2c2f\left( X_{P_4\text {-}Down}\right) \end{aligned} \end{aligned}$$

Where $X_{P_4{-}Down}$ represents the intermediate features after $P_4$ downsampling and semantic enhancement; ${MaxPool}(\cdot )$ denotes the max pooling operation, employing a *2× 2* pooling kernel, stride *s=2*, and zero padding to reduce spatial resolution while preserving the most prominent semantic information within the features; *DSConv(· )* denotes Depthwise Separable Convolution, which extracts spatial features channel-wise to enhance the representation of lesion edges and local structural details; *PointwiseConv(· )* performs pointwise convolution using a *1× 1* kernel, stride *s=1*, and zero padding , fusing single-channel features extracted from deep convolutions to establish semantic correlations between channels; *A2c2f(· )* is the adaptive channel-to-feature fusion module.

$P_5$ represents the top-level deep feature, carrying the most abstract global anatomical semantics such as the overall distribution of lung lobes and the macroscopic anatomical localization of lesions. It provides high-level semantic constraints for the global search-shape perception process of minute lesions. The computational process is shown in Equation (14).

14 $$\begin{aligned} P_5 = \text {A2c2f}\left( \text {CEFEN}\left( \text {DSConv}\left( \text {MaxPool}(P_4) \right) \right) \right) \end{aligned}$$

Among these, *MaxPool(· )* represents the subsampled features from $P_4$, employing a *2× 2* pooling kernel with stride *s=2* to further reduce spatial dimensions and emphasize global semantic information; $P_5$ denotes the feature map from the global semantic support layer.

### Adaptive feature focusing network

**Figure 3:**
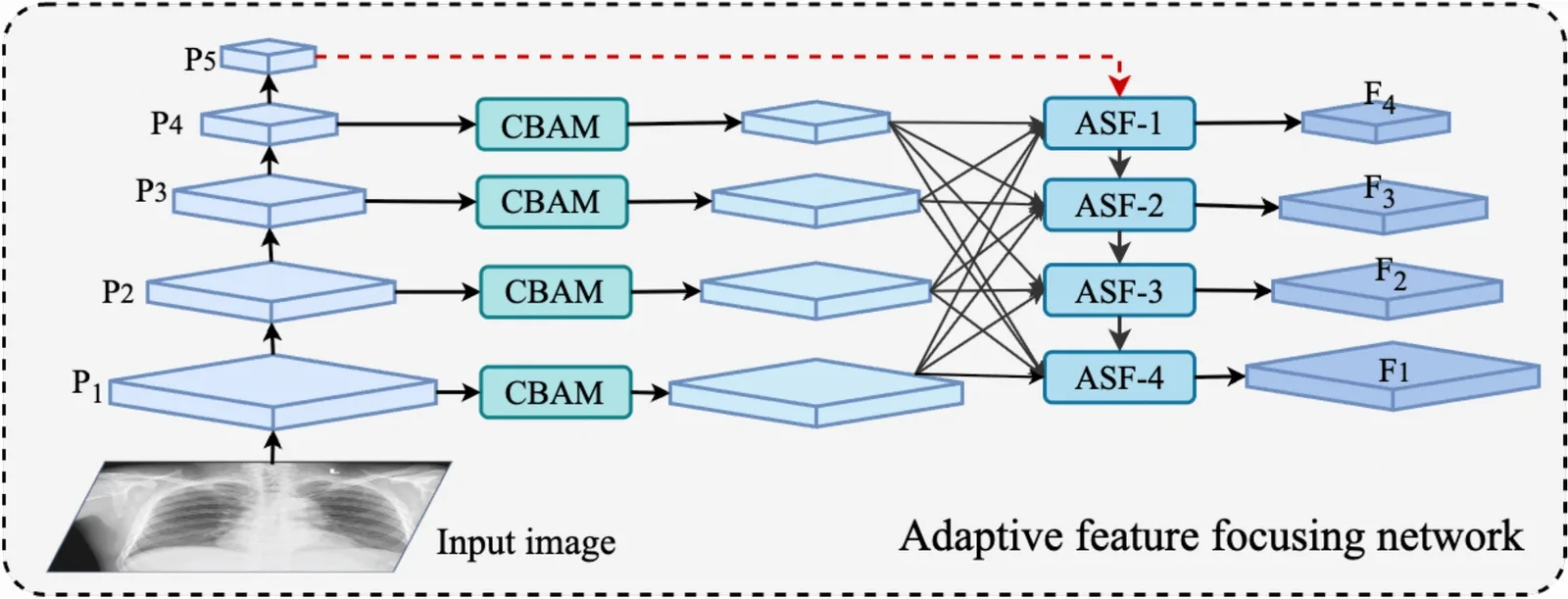
Adaptive feature focusing network architecture diagram.

Due to significant variations in lesion size, morphology, and contrast within chest images, traditional feature pyramid structures often suffer from semantic misalignment and loss of fine-grained details when fusing multi-level features. This leads to degraded model performance in detecting small lesions and identifying features within complex backgrounds. To address this, the Adaptive Feature Focusing Network (AFFN) builds on the AFPN architecture to establish an attention-guided, multi-scale feature-focusing framework, as illustrated in Fig. 3. Through CBAM feature enhancement and the Adaptive Spatial Fusion (ASF) mechanism, it achieves dynamic focusing and refined multi-scale feature fusion.

Input images undergo feature extraction through the backbone network to yield multi-level features ${P_1, P_2, P_3, P_4, P_5}$. To enhance the distinctiveness and discriminative power of each feature layer, AFFN embeds a Convolutional Block Attention Module (CBAM) at every level for feature reinforcement. CBAM employs a cascaded operation of channel attention and spatial attention to effectively suppress redundant background information and highlight regions relevant to lesions. This process endows each feature map with stronger discriminative capability, providing high-quality input for subsequent multi-scale fusion. The computational process is illustrated in Equation (15).

15 $$\begin{aligned} \begin{aligned} M_c(\tilde{P}_j)&= \sigma \left( \text {MLP}\left( \text {AvgPool}(\tilde{P}_j) \right) + \text {MLP}\left( \text {MaxPool}(\tilde{P}_j) \right) \right) \\ \check{P}_j&= M_c(\tilde{P}_j) \odot \tilde{P}_j \\ M_s(\check{P}_j)&= \sigma \left( \text {Conv}_{7\times 7}\left( \left[ \text {AvgPool}_{ch}(\check{P}_j); \text {MaxPool}_{ch}(\check{P}_j) \right] \right) \right) \\ \dot{P}_j&= M_s(\check{P}_j) \odot \check{P}_j \end{aligned} \end{aligned}$$

Where $\tilde{P}_j$ denotes the feature map aligned in size and channels via upsampling or downsampling, *j ∈ [1,4]*; $M_c(\cdot )$ represents channel attention; *AvgPool(· )* and *MaxPool(· )* correspond to average pooling and max pooling within channel attention, respectively; *MLP(· )* denotes the fully connected layer; $\sigma (\cdot )$ represents the sigmoid function; $\check{P}_j$ is the feature map weighted by the channel attention; *⊙* denotes element-wise multiplication; $M_s (\cdot )$ denotes the spatial attention; $AvgPool_{ch}(\cdot )$ and $MaxPool_{ch}(\cdot )$ denote spatial attention’s average pooling and max pooling over channels, respectively; $Conv_(7\times 7)$ represents the *7× 7* convolution operation using a *7× 7* convolution kernel; $[\cdot ,\cdot ]$ denotes concatenation; $\dot{P}_j$ denotes the feature map output after CBAM enhancement.

Due to the unidirectional top-down information flow in traditional FPNs, deep-level semantic information gradually degrades during propagation to lower layers. Therefore, a progressive fusion strategy combining top-down and lateral connections is adopted. High-level semantic features propagate downward through subsampling paths to provide semantic guidance for local regions. Simultaneously, low-level features supplement high-level spatial details via lateral connections, enabling bidirectional interaction between semantic and spatial information. The top-down semantic guidance computation process is illustrated in Equation(16).

16 $$\begin{aligned} \begin{aligned} G_5&= \text {Proj}(P_5) \\ L_i&= \text {Proj}(\dot{P}_j) \\ \tilde{G}_i&= \text {Upsample}(G_{i+1}) + L_i \\ G_i&= \text {Act}\left( \text {Conv}_{3\times 3}(\tilde{G}_i) \right) \\ \tilde{F}_i&= G_i + \gamma _i \cdot \dot{P}_j \end{aligned} \end{aligned}$$

Where $G_5$ represents the projection of feature map $P_5$; *Proj(· )* denotes a *1× 1* convolution kernel followed by a Batch Normalization (BN) operation, aligned with the channel; $L_i$ denotes the projection of feature map $\dot{P}_j$; *Act(· )* denotes a convolution followed by BN and ReLU operations; $\tilde{G}_i$ denotes the feature map of layer i output from the progressive focusing stage; $G_i$ denotes the refined semantic representation after top-down and lateral fusion; $\gamma _i$ represents the learnable scaling coefficient; $\tilde{F}_i$ denotes the intermediate feature map.

To achieve spatially adaptive dynamic fusion across different feature levels and enhance the model’s ability to detect multi-scale lesions–tiny lesions–an Adaptive Spatial Fusion (ASF) attention mechanism is introduced. During multi-level feature aggregation, ASF assigns adaptive weights to each spatial location, enabling the model to automatically adjust the contributions of features across hierarchical levels based on lesion characteristics.

First, perform a lightweight transformation to align channels for each target output scale i (corresponding to ${ASF-}i$). Adaptively aggregate information from all source scales j, and compute learnable weights at spatial positions (*x*, *y*) to balance residual contributions. The computation process is shown in Equation (17).

17 $$\begin{aligned} \begin{aligned} Q_j&= \text {Proj}\left( \text {Resize}(\tilde{F}_i; H, W) \right) \\ S_{ij}(x,y)&= \phi _i\left( Q_j(x,y) \right) \\ W_{ij}(x,y)&= \frac{\exp \left( S_{ij}(x,y) \right) }{\sum _{K \in S} \exp \left( S_{iK}(x,y) \right) } \end{aligned} \end{aligned}$$

Where $Q_j$ denotes the feature map after dimensional and channel alignment; *Resize(· )* refers to the operation that unifies the resolution to *H× W* and the channel count to *C*; $S_ij (x,y)$ represents the position-level scoring map computed for each scale i and its source j; $\phi _i$ denotes the scoring subnetwork for ${ASF-}i$; $W_ij (x,y)$ denotes the weight applied to $S_{ij} (x,y)$ for Softmax normalization.

Perform a linear combination of the aligned source vectors using the obtained positional weights to obtain the vector representation of the target layer’s output features at position (*x*, *y*), as shown in Equation (18).

18 $$\begin{aligned} \dot{F}_i = \sum _{j \in S} W_{ij} \cdot U_{ij}(Q_j) \end{aligned}$$

Where $U_{ij}(\cdot )$ is the identity mapping, further aligning the channels; *j ∈ S* denotes features at different scales, $S={1,2,3,4}$; $\dot{F}_i$ represents the layer-level output features after ASF fusion.

Finally, introducing $P_5$’s global semantic compensation, we perform residual fusion between $P_5$ and $\dot{F}_i$ to output a representation that preserves global contextual semantics, as shown in Equation (19).

19 $$\begin{aligned} F_i = \dot{F}_i + \alpha \cdot G_5 \end{aligned}$$

Where *α* is a learnable scalar; $F_i$ represents the final output features $(F_1, F_2, F_3, F_4)$ of the AFFN. By integrating the global semantics from fusion $P_5$, it significantly enhances scale adaptability and localization accuracy for small lesions, directly feeding into subsequent detection heads for lesion classification and bounding box regression.

### Shape perception optimization strategy loss function

Traditional bounding box regression loss functions, such as IoU, GIoU, DIoU, or CIoU, primarily measure similarity between target and predicted boxes based on overlapping regions. However, when lesions are small, irregularly shaped, or slightly misaligned, these region-based metrics exhibit unstable gradients, resulting in insufficient sensitivity for locating small lesions. To address this, this paper proposes a Shape-Aware Optimization Strategy (SAOS). By integrating Normalized Wasserstein Distance (NWD) with shape-weighted Medical IoU (MSIoU) into a unified model, SAOS enhances the model’s sensitivity to geometric variations while maintaining scale invariance.

First, calculate the normalized Wasserstein distance for spatial distribution modeling. Let the predicted bounding box and the ground-truth bounding box be defined as shown in Equation (20).

20 $$\begin{aligned} \begin{aligned} B_p&= (x_p, y_p, w_p, h_p), \\ B_g&= (x_g, y_g, w_g, h_g). \end{aligned} \end{aligned}$$

Where $B_p$ denotes the predicted boundary box, while $B_g$ represents the true bounding box corresponding to the lesion in the data. Both boxes contain center coordinates (*x*, *y*), with *w* and *h* denoting width and height, respectively.

To enhance the continuity of spatial alignment, both the predicted bounding box and the true bounding box are modeled as two-dimensional Gaussian distributions, as shown in Equation (21).

21 $$\begin{aligned} \begin{aligned} \mathscr {N}_p&\sim \mathscr {N}(\mu _p, \Sigma _p) \\ \mathscr {N}_g&\sim \mathscr {N}(\mu _g, \Sigma _g) \\ \mu&= (x, y) \\ \Sigma&= \text {diag}\left[ \left( \frac{w}{2} \right) ^2, \left( \frac{h}{2} \right) ^2 \right] \end{aligned} \end{aligned}$$

Where $\mu _p$ and $\mu _g$ denote the mean values of the Gaussian distribution; $\Sigma _p$ and $\Sigma _g$ represent the covariance matrices, characterizing the spatial extent of the boxes; $\mathscr {N}_p$ and $\mathscr {N}_g$ denote the Gaussian representations of the predicted and ground-truth bounding boxes, respectively.

Thus, the second-order Wasserstein distance between the two bounding-box distributions is calculated as shown in Equation (22).

22 $$\begin{aligned} W_2^2(\mathscr {N}_p, \mathscr {N}_g) = \Vert \mu _p - \mu _g\Vert _2^2 + \left\| \Sigma _p^{1/2} - \Sigma _g^{1/2} \right\| _F^2 \end{aligned}$$

Where $W_2^2(\cdot )$ denotes the second-order Wasserstein distance, which measures the overall discrepancy between two Gaussian distributions in terms of spatial location and shape; $\Vert \cdot \Vert _2^2$ denotes the squared Euclidean norm, which computes the squared distance between two vectors and quantifies the difference in the center positions; $\Vert \cdot \Vert _F^2$ d denotes the squared Frobenius norm, which is used to measure matrix discrepancies and capture differences in the shape of the bounding box in terms of width and height.

To accommodate targets of varying scales, the normalized Wasserstein similarity is defined as shown in Equation (23).

23 $$\begin{aligned} \text {NWD}(B_p, B_g) = \exp \left( -\frac{W_2^2(\mathscr {N}_p, \mathscr {N}_g)}{2\sigma ^2} \right) \end{aligned}$$

Where *σ* is the scale normalization coefficient, which controls the decay rate of similarity to mitigate the vanishing gradient problem in IoU-based losses during small object detection. ${NWD}(B_p, B_g)$ represents the normalized Wasserstein distance, measuring the spatial consistency between predicted and ground-truth bounding boxes, with a value range of [0, 1].

Lesions in medical images exhibit substantial shape variability, and their boundaries are often affected by surrounding anatomical structures. To enhance the model’s sensitivity to morphological differences, a shape-adaptive weighting term $\omega _s$ is introduced on top of IoU, jointly considering aspect ratio consistency, area proportion consistency, and boundary structure consistency. The corresponding formulation is given in Equation (24).

24 $$\begin{aligned} \begin{aligned} \Delta _r&= 1 - \frac{\min (\omega _p/h_p, \omega _g/h_g)}{\max (\omega _p/h_p, \omega _g/h_g)} \\ \Delta _a&= 1 - \frac{\min (\omega _p h_p, \omega _g h_g)}{\max (\omega _p h_p, \omega _g h_g)} \\ \Delta _b&= \frac{1}{H \times W} \left| \nabla I_p(x,y) - \nabla I_g(x,y) \right| \\ \omega _s&= \exp (-\eta _r \Delta _r^2) \cdot \exp (-\eta _a \Delta _a^2) \cdot \exp (-\eta _b \Delta _b^2) \end{aligned} \end{aligned}$$

Where $\Delta _r$ represents the aspect ratio difference, reflecting the consistency between the predicted box and the ground truth box in terms of width-to-height ratio; $\Delta _a$ denotes the area ratio difference, measuring the relative deviation in area between the predicted box and the ground truth box; $\Delta _b$ denotes the boundary gradient difference, characterizing the structural discrepancy between the predicted lesion and the ground truth lesion; $\nabla I_p(x,y)$ and $\nabla I_g(x,y)$ represent the lesion region images used to compute the boundary gradient difference $\Delta _b$; H and W denote the image height and width, respectively, used for gradient normalization; $\omega _s$ is the shape weighting factor, representing the dynamic weighting that comprehensively considers the aspect ratio difference, area ratio, and boundary difference; $\eta _r, \eta _a$, and $\eta _b$ are the shape weighting coefficients, controlling the proportional weighting of different geometric indicators within $\omega _s$.

When the predicted box is close in shape to the ground truth box, $\omega _s$ approaches 1; otherwise, it decays exponentially. Medical shape-weighted IoU is calculated as shown in as shown in Equation (25).

25 $$\begin{aligned} \text {MSIoU}(B_p, B_g) = \omega _s \cdot \text {IoU}(B_p, B_g) \end{aligned}$$

Where *IoU* denotes the intersection-over-union ratio, representing the ratio of the overlapping area between predicted and ground-truth bounding boxes to their combined area. *MSIoU* is the medical shape-weighted IoU, which incorporates a shape weighting factor $\omega _s$ on top of *IoU* to account for morphological variations. Unlike CIoU or EIoU, MSIoU maintains effective gradient constraints even for lesions with significant shape variations or irregular boundaries.

By integrating spatial distribution similarity with geometric shape constraints, we define the final shape-aware loss function (SAOS Loss), as shown in Equation (26).

26 $$\begin{aligned} \mathscr {L}_{SAOS} = 1 - \left( \lambda _1 \cdot \text {MSIoU}(B_p, B_g) + \lambda _2 \cdot \text {NWD}(B_p, B_g) \right) \end{aligned}$$

Where $\mathscr {L}_{SAOS}$ denotes the shape-aware loss function; $\lambda _1$ and $\lambda _2$ represent the SAOS weighting coefficients, which control the relative importance of *MSIoU* and *NWD* in the final loss.

## Experiments and results

### Experiment setup

The software environment for this experiment comprises the Torch 2.0.0 framework, CUDA 11.8, Python 3.8.10, torchvision 0.15.1, OpenCV 4.9.0.88, mmcv 2.1.0, and mm-detection 3.3.0. The experimental hardware environment utilizes a single NVIDIA RTX 4090 GPU. During training, standard random data augmentation operations–including scaling, flipping, and translation–were applied to expand the training dataset. Experimental parameters were set as follows: batch size is 16 and training epochs is 400. The AdamW optimizer was employed to regulate the learning rate, with an initial learning rate of 0.001, *β* parameters (0.9, 0.999), and weight decay of 0.001.

### Evaluation metrics

To comprehensively evaluate the performance of the proposed model in multi-disease detection tasks, Precision, Recall, *mAP*@0.5, *mAP*@[0.5 : 0.95], $AP_S$, $AP_M$, and $AP_L$ were adopted as evaluation metrics. Here, $AP_S$, $AP_M$, and $AP_L$ denote the average precision for small, medium, and large lesions, respectively. Following the common COCO-style evaluation protocol, small lesions are defined as objects with an area smaller than 32*×*32 pixels, medium lesions as objects with an area between 32*×*32 and 96*×*96 pixels, and large lesions as objects with an area larger than 96*×*96 pixels. Among these, *mAP* serves as a core evaluation metric in the object detection domain. Its calculation relies on Intersection over Union (IoU), which quantifies the spatial overlap between predicted and ground-truth bounding boxes by computing the ratio of their overlapping area to their combined area, as shown in Equation(27).

27 $$\begin{aligned} \text {IoU}(A, B) = \frac{\text {Area}(A \cap B)}{\text {Area}(A \cup B)} \end{aligned}$$

Where *A* denotes the predicted bounding box position, and *B* denotes the annotated bounding box position. In this experiment, the IoU threshold is set to 0.5.

Precision and Recall are commonly used metrics to reflect a model’s accuracy and recognition capability when processing positive and negative samples. Positive samples represent instances where lesions are present in the image, while negative samples indicate instances where no task-relevant lesions exist in the image. Precision measures the proportion of instances correctly classified as positive by the model that are actually positive samples. Recall, conversely, indicates the proportion of all true positive samples that are correctly identified by the model. Their calculation processes are shown in Equation (28).

28 $$\begin{aligned} \begin{aligned} \text {Precision}&= \frac{TP}{TP + FP} \\ \text {Recall}&= \frac{TP}{TP + FN} \end{aligned} \end{aligned}$$

Where *TP* represents true positives, indicating the number of actual lesions correctly detected; *FP* denotes false positives, representing the number of negative samples or background areas misclassified as lesions; *FN* signifies false negatives, reflecting the number of actual lesions that were not detected.

To comprehensively evaluate a model’s detection performance across different task conditions, *mAP* calculates the area under the precision-recall curve at multiple IOU thresholds. The mean average precision $\text {mAP}@0.5$ is computed at an IOU threshold of 0.5 and serves as a benchmark for evaluating general object detection tasks. $\text {mAP}@[0.5:0.95]$, however, is the average of the mean average precision (AP) calculated across multiple IoU thresholds ranging from 0.5 to 0.95 with a step size of 0.05. It is suitable for tasks demanding higher localization accuracy. Its calculation process is shown in Equation (29).

29 $$\begin{aligned} \begin{aligned} \text {AP}&= \int _{0}^{1} P(r) dr \\ \text {mAP}&= \frac{1}{N} \sum _{i=1}^{n} \text {AP}_i \end{aligned} \end{aligned}$$

Where *P*(*r*) denotes the precision as a function of recall; $AP_i$ represents the average precision of the i-th class; and *N* denotes the total number of classes, which is set to 14 in this experiment.

### Dataset

Currently available publicly accessible chest multi-disease datasets include CheXpert, ChestX-ray14, MIMIC-CXR, VinDr-CXR^36^, and ChestX-ray8^37^. Among these datasets, VinDr-CXR provides high-quality bounding-box annotations manually labeled by experienced radiologists and is suitable for evaluating multi-disease localization performance in chest X-ray images. To further assess the generalization capability of the proposed method, ChestX-ray8 was also introduced as an additional validation dataset. Therefore, experiments were conducted on both VinDr-CXR and ChestX-ray8 in this study.

**Figure 4:**
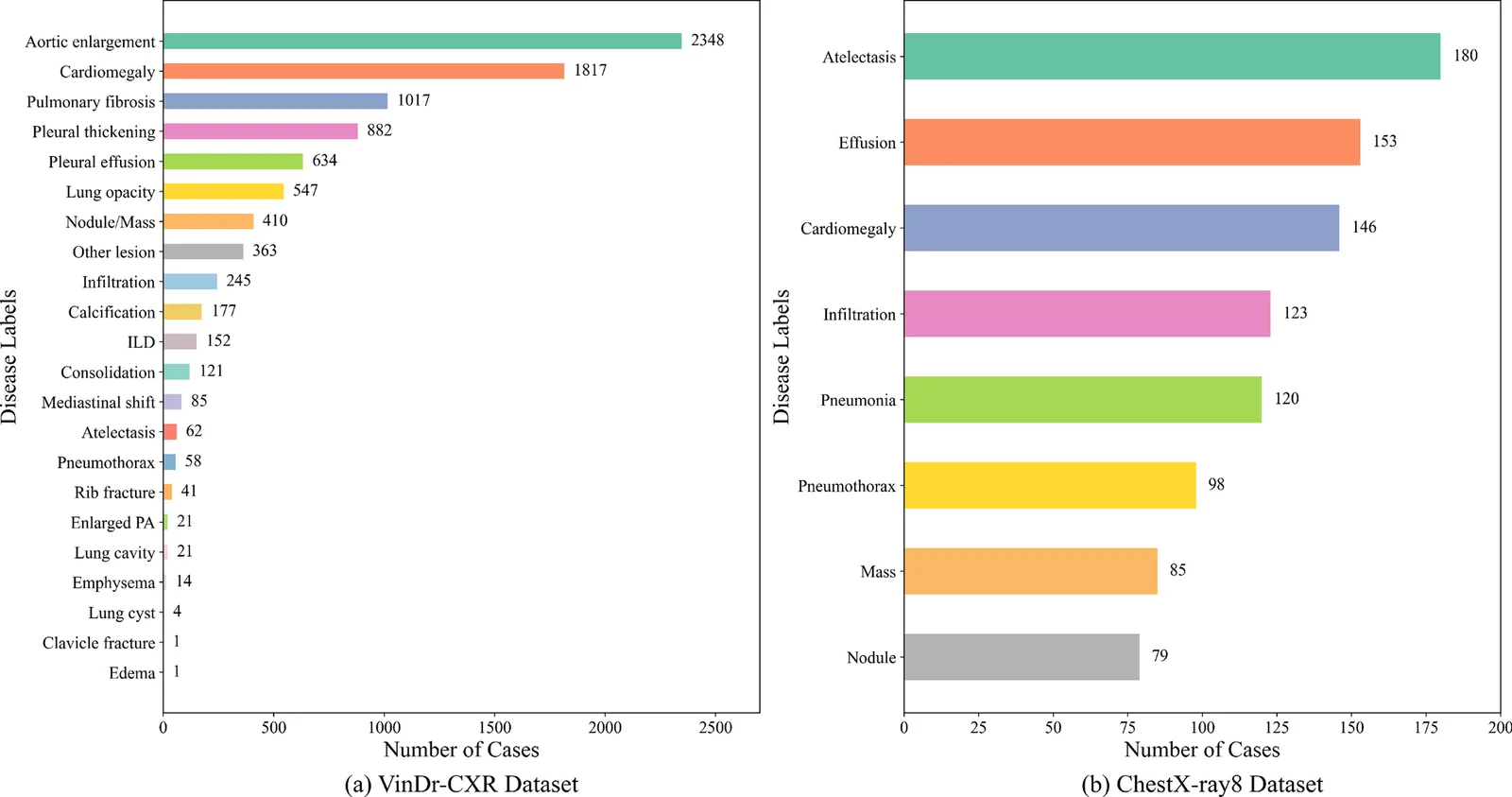
Disease category distributions of the VinDr-CXR and ChestX-ray8 datasets. (**a**) Distribution of disease labels in the VinDr-CXR dataset. (**b**) Distribution of disease labels in the ChestX-ray8 dataset.

As shown in Fig. 4, the disease category distributions of VinDr-CXR and ChestX-ray8 exhibit different characteristics. The VinDr-CXR dataset consists of a training set containing 15,000 images and a test set containing 3000 images. It provides classification and localization annotations for 22 disease categories, which were manually labeled by 17 experienced radiologists, and is specifically designed for chest disease classification and localization in X-ray images. As shown in Fig 4(a), VinDr-CXR presents a clear long-tailed distribution, with several categories such as Aortic enlargement, Cardiomegaly, and Pulmonary fibrosis containing relatively more samples, while some abnormal categories contain only a small number of samples. Due to this severe class imbalance, 500 images from the normal category were randomly selected, and the eight disease categories with the smallest number of samples were merged into a single category referred to as “Other lesion” to stabilize network training.

Compared with VinDr-CXR, ChestX-ray8 exhibits different data distribution and annotation characteristics. As shown in Fig. 4(b), several findings, such as Atelectasis, Effusion, Cardiomegaly, Infiltration, and Pneumonia, have relatively more balanced representation in the localization subset used in this study. The available bounding-box annotations of ChestX-ray8 were used without additional annotation fusion. All images were resized to 512 *×* 512 pixels, and a random 9:1 split was adopted for training and testing. By conducting experiments on both VinDr-CXR and ChestX-ray8, the proposed method can be evaluated under different data distributions, annotation settings, and lesion category characteristics, providing a more comprehensive assessment of its effectiveness and robustness in multi-disease chest lesion detection.

### Data augmentation

**Figure 5:**
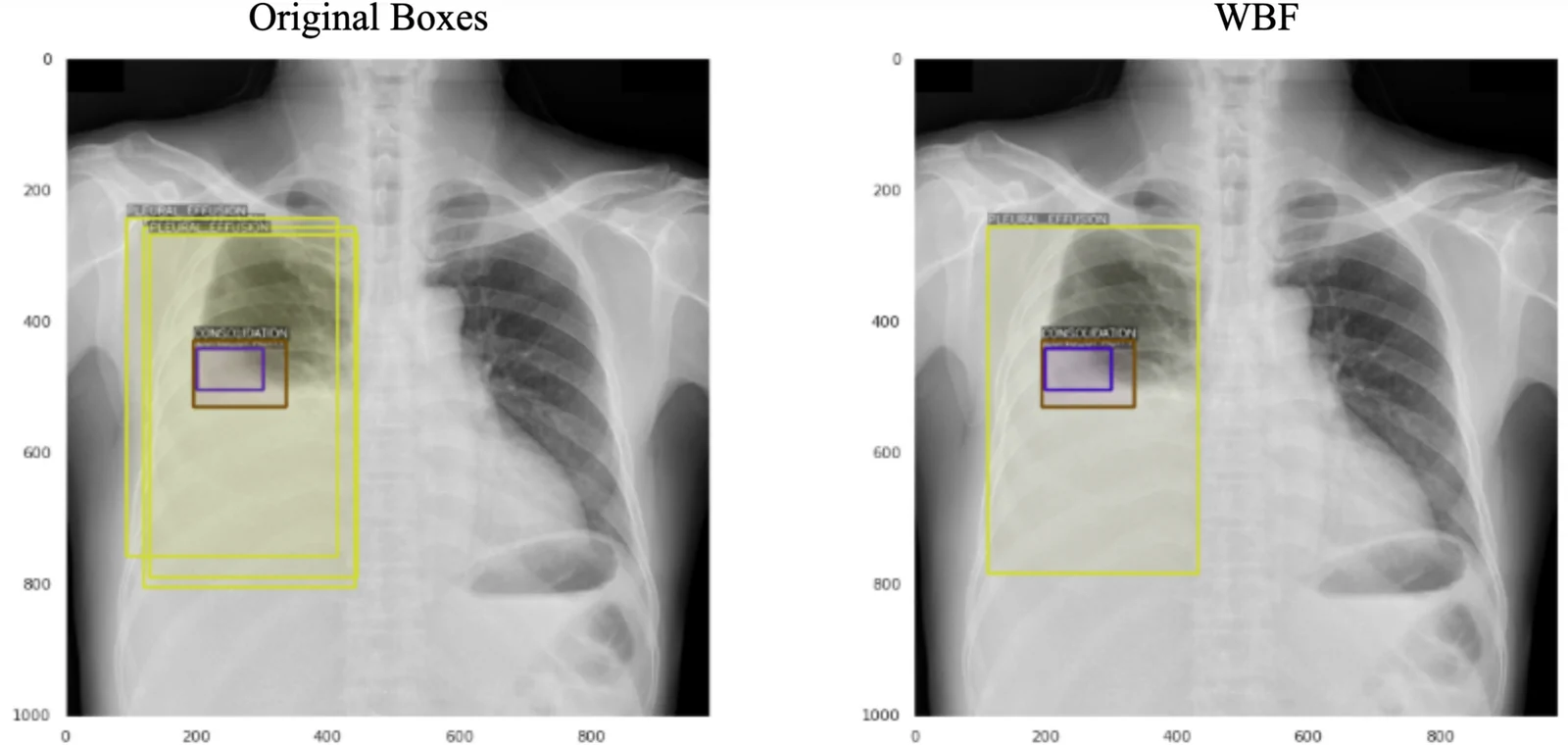
Comparison between the original labeled bounding box and the bounding box after WBF processing.

Since the chest images in VinDr-CXR were annotated independently by 14 professional radiologists, numerous overlapping annotation bounding boxes appear in the dataset images, potentially making it difficult for the model to correctly identify and learn the target boundaries. Therefore, weighted box fusion (WBF) technology^38^ was employed to reduce annotation box overlap and redundancy. As shown in Fig. 5, the left image displays the original annotated label bounding boxes, while the right image shows the label bounding boxes after WBF application. Following WBF preprocessing, the annotation boxes are significantly clearer and more accurate.

### Results

To validate the effectiveness and generalization capability of the proposed method, experiments were conducted on two public chest X-ray datasets, namely VinDr-CXR and ChestX-ray8, and the proposed method was compared with several state-of-the-art detectors. To ensure a fair comparison, all compared methods were trained and evaluated under a unified experimental protocol, as summarized in Table 1. Specifically, all input images were resized to 512 *×* 512 pixels, and the same training schedule, batch size, optimizer, initial learning rate, evaluation metrics, and hardware environment were used for all methods. For dataset-specific preprocessing, Weighted Box Fusion (WBF) was applied to the VinDr-CXR annotations to reduce redundant bounding boxes generated by multiple radiologists, whereas the original annotations of ChestX-ray8 were used without additional fusion. The official train/test split was adopted for VinDr-CXR, while a random 9:1 split was used for ChestX-ray8. These unified settings ensure that the performance differences mainly result from model design rather than inconsistent implementation details or training configurations.

**Table 1:** Unified experimental settings for all compared methods on the VinDr-CXR and ChestX-ray8 datasets.

Item	Unified configuration
Dataset	VinDr-CXR; ChestX-ray8
Input resolution	*512× 512* pixels
Annotation processing	VinDr-CXR: Weighted Box Fusion (WBF); ChestX-ray8: original annotations without additional fusion
Train/Test split	VinDr-CXR: official split; ChestX-ray8: random 9:1 split
Training epochs	400
Batch size	16
Optimizer	AdamW (weight decay = 0.001)
Initial learning rate	0.001
Evaluation metrics	*mAP*@0.5, *mAP*@[0.5 : 0.95], $AP_S$, $AP_M$, $AP_L$, Recall, Params (M)
Hardware	Single NVIDIA RTX 4090; PyTorch 2.0; CUDA 11.8

#### Comparison on VinDr-CXR dataset

**Figure 6:**
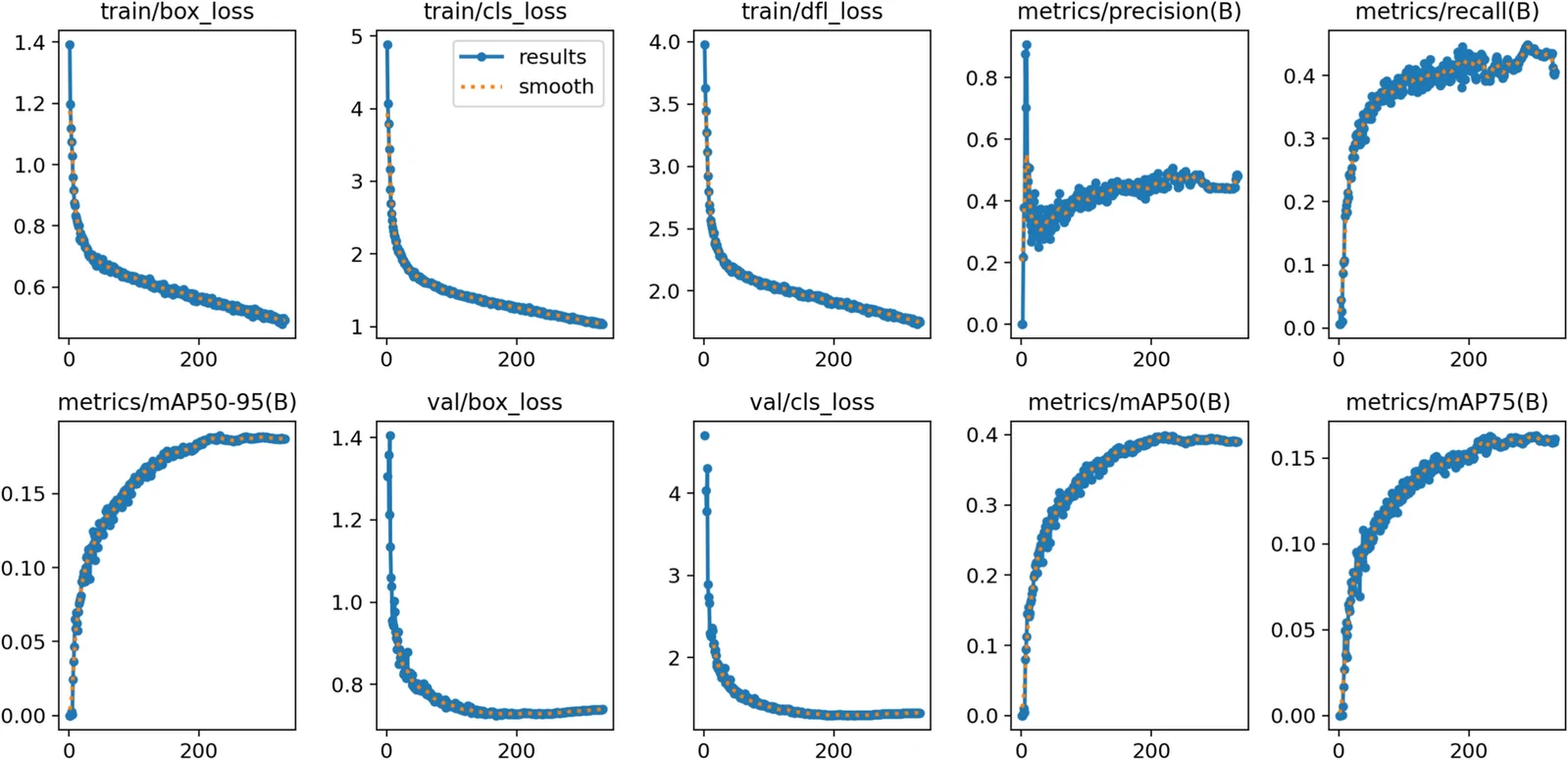
Training and validation curves of loss functions, precision, recall, and mean average precision (*mAP*).

As shown in Fig. 6, all loss functions in the proposed method exhibit excellent convergence characteristics during training. The box loss, cls loss, and DFL loss rapidly decrease in the initial stages of training before gradually stabilizing, indicating that the model effectively learns the discriminative features and localization information of the targets. The overall optimization process remains stable without significant oscillations. Simultaneously, the box loss and cls loss on the validation set follow consistent trends with the training phase, showing no significant rebound in the later stages of training. This demonstrates that the model maintains strong generalization capabilities under complex data distribution conditions and does not exhibit noticeable overfitting.

In terms of detection performance metrics, Precision, Recall, and mAP consistently improved throughout training iterations before eventually stabilizing. The Recall curve maintained a high growth trend in the mid-to-late stages, indicating the model progressively reduced false negatives. Simultaneously, Precision remained stable in the later stages, demonstrating that performance gains did not come at the cost of significantly increased false positives. *mAP*@0.5, and *mAP*@[0.5 : 0.95] all exhibited smooth upward trajectories before converging, reflecting the model’s robust detection and localization capabilities across different IoU thresholds. Overall results demonstrate that the proposed method exhibits excellent training stability, consistent performance gains, and system robustness, showcasing strong potential for practical applications.

**Figure 7:**
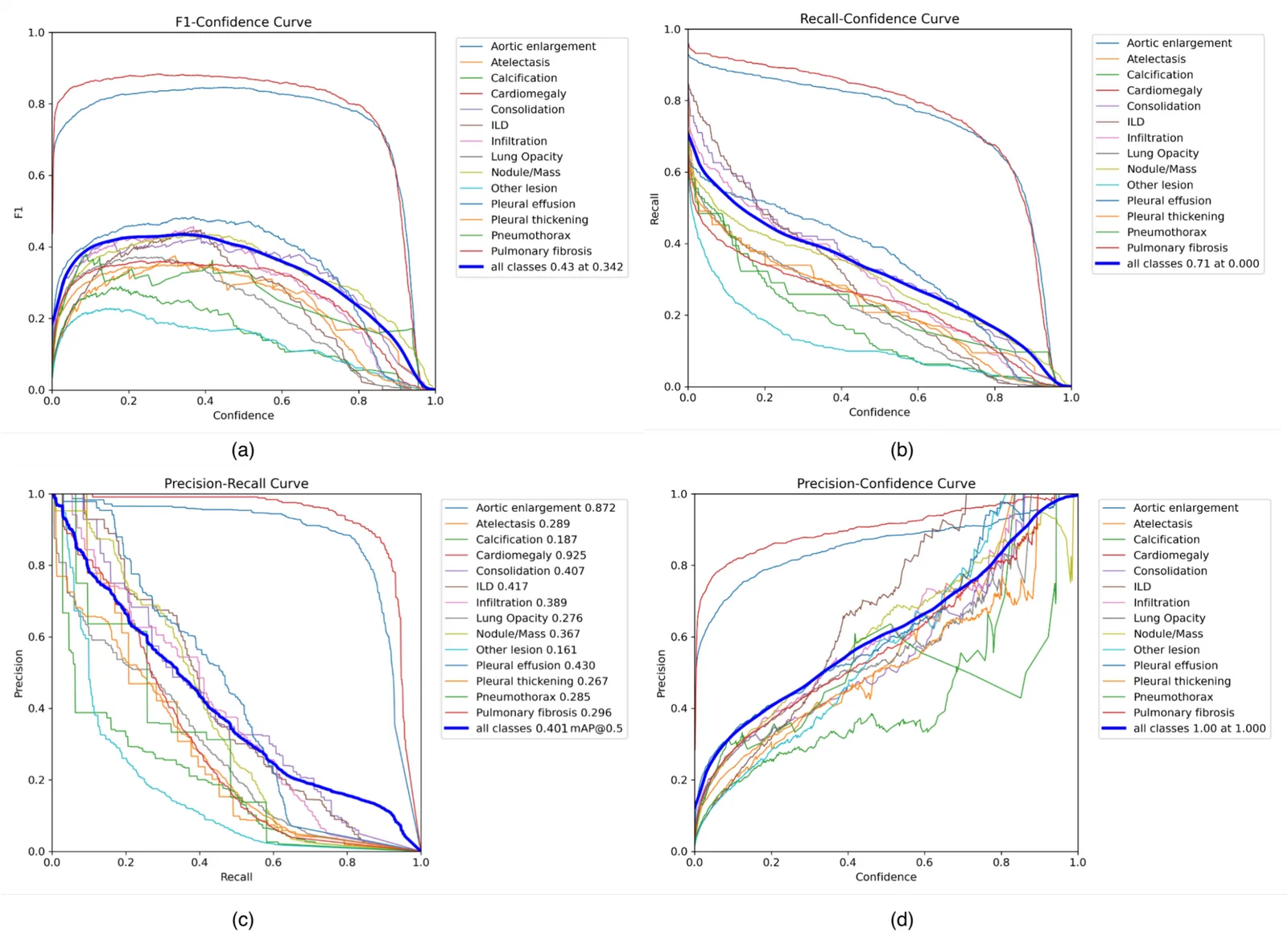
Confidence-based performance curves of the proposed method. (**a**) F1-confidence curves; (**b**) recall-confidence curves; (**c**) precision-recall curves; (**d**) precision-confidence curves.

Furthermore, the detection performance of the model was statistically analyzed using the F1-confidence, precision-confidence, recall-confidence, and precision-recall curves shown in Fig. 7. As shown in Fig. 7(a), under different conditions, the F1 score gradually increases within the lower confidence interval as the threshold rises, peaks at a moderate confidence level, and then gradually decreases as confidence further increases. This trend indicates that at lower confidence thresholds, a more reasonable balance between positive and negative samples can be achieved, thereby demonstrating superior overall detection capability.

As shown in Fig. 7(b), even at lower confidence levels, the model maintains high precision, indicating its strong discriminative capability in target recognition and effective suppression of false positives. Simultaneously, Fig. 7(c) reveals that recall gradually decreases as the confidence threshold increases. This reflects the model’s trade-off: while enhancing prediction reliability, it inevitably sacrifices some detection capability. Further analysis of the precision-recall curve in Fig. 7(d) reveals that the model maintains relatively high precision across a broad range of recall values. This demonstrates stable detection performance and robust adaptability across different decision threshold settings, making it suitable for flexible deployment in practical intelligent detection systems.

The experimental results are shown in Table 2. This method achieves state-of-the-art performance on key metrics including *mAP*@0.5, *mAP*@[0.5 : 0.95], detection capabilities across different scales (APs, APm, APl), and Recall, while maintaining high efficiency in terms of model parameter size.

**Table 2:** Performance comparison with state-of-the-art methods on the VinDr-CXR dataset.

Model	mAP@0.5	*mAP*@[0.5 : 0.95]	$AP_S$	$AP_M$	$AP_L$	Params (M)	Recall
RefineDet^39^	0.128	0.075	–	–	–	33.51	–
SAR-CNN^40^	0.158	0.119	0.065	0.290	0.357	56.48	0.253
EARL^41^	0.208	0.143	0.072	0.266	0.370	55.24	0.238
DualAttNet^42^	0.243	0.116	0.005	0.198	0.121	–	0.257
RT-DETR^43^	0.266	0.149	–	–	–	32.01	–
YOLO-CXR^23^	0.338	0.167	0.095	0.313	0.416	–	0.365
Ours	0.401	0.196	0.159	0.361	0.466	25.0	0.451

In terms of overall detection accuracy, the proposed method achieves the best performance, reaching values of 0.401 for *mAP*@0.5 and 0.196 for *mAP*@[0.5 : 0.95], which are markedly superior to those of existing approaches. When compared with the strong baseline YOLO-CXR, which attains scores of 0.338 and 0.167 on the same metrics, the proposed method yields absolute improvements of 6.1% and 2.9%, respectively. The notable gain observed under the more stringent *mAP*@[0.5 : 0.95] metric indicates that the proposed approach not only improves lesion detection capability but also provides more stable localization accuracy at higher IoU thresholds. This property is particularly important for chest imaging scenarios involving lesions with blurred boundaries and irregular morphologies.

Regarding detection performance across different lesion scales, the proposed method consistently delivers state-of-the-art results on small, medium, and large-scale lesions. The detection accuracy for small lesions reaches 0.159, substantially exceeding that of SAR-CNN, EARL, and YOLO-CXR, demonstrating a clear advantage in identifying minute lesions that constitute a large proportion of chest images and carry high diagnostic significance. This performance improvement is closely aligned with the design objectives of the proposed context-embedded feature enhancement and adaptive feature focusing mechanisms, which are specifically intended to strengthen fine-grained feature representation. Meanwhile, the stable performance gains achieved for medium- and large-scale lesions, with scores of 0.361 and 0.466, indicate that the optimization for small targets does not compromise detection capability at other scales, reflecting strong scale generalization.

With respect to recall performance, the proposed method attains the highest value of 0.451, showing a clear improvement over YOLO-CXR, which achieves 0.365, and DualAttNet, which reaches 0.257. The enhanced recall demonstrates that the model effectively reduces the risk of missed detections under complex anatomical conditions. This property is especially important in clinical diagnostic assistance scenarios, where missed detections generally pose greater potential risks than false positives.

While delivering consistent performance improvements across multiple evaluation metrics, the proposed method maintains a relatively compact model size of 25.0 million parameters. This parameter count is substantially lower than those of SAR-CNN with 56.48 million parameters, EARL with 55.24 million parameters, and RT-DETR with 32.01 million parameters. These results indicate that the observed performance gains are not achieved through simple model scaling, but rather through more efficient feature representation and optimized architectural design, resulting in a favorable tradeoff between performance and model complexity.

**Table 3:** Comparison of average precision with state-of-the-art methods across 14 disease categories on the VinDr-CXR dataset.

Disease labels	DualAttNet^42^	EARL^41^	YOLOv9^44^	YOLO-CXR^23^	Ours
Aortic enlargement	0.634	0.549	0.877	0.885	0.883
Atelectasis	0.153	0.149	0.181	0.235	**0.275**
Calcification	0.061	0.085	0.053	0.114	**0.205**
Cardiomegaly	0.671	0.566	0.903	0.912	**0.926**
Consolidation	0.199	0.139	0.309	0.327	**0.387**
ILD	0.255	0.281	0.203	0.234	**0.411**
Infiltration	0.232	0.185	0.282	0.292	**0.395**
Lung Opacity	0.155	0.154	0.150	0.196	**0.293**
Nodule/Mass	0.140	0.137	0.199	0.208	**0.348**
Other lesion	0.295	0.052	0.343	0.404	0.174
Pleural effusion	0.189	0.252	0.136	0.175	**0.447**
Pleural thickening	0.142	0.104	0.336	0.391	0.280
Pneumothorax	0.197	0.123	0.261	0.262	**0.267**
Pulmonary fibrosis	0.082	0.144	0.055	0.095	**0.309**
*mAP*@0.5	0.243	0.208	0.306	0.338	**0.401**
*mAP*@[0.5 : 0.95]	0.116	0.143	0.156	0.167	**0.196**
Recall	0.257	0.238	0.325	0.365	**0.451**

Table 3 presents the quantitative comparison between the proposed method and several state-of-the-art approaches, including DualAttNet, EARL, YOLOv9, and YOLO-CXR on the VinDr-CXR dataset. The evaluation metrics include per-class Average Precision (AP), *mAP*@0.5, *mAP*@[0.5 : 0.95], and recall. In our study, the proposed method achieved the best overall performance among all compared approaches. Specifically, our method obtained an *mAP*@0.5 of 0.401, which was higher than DualAttNet (0.243), EARL (0.208), YOLOv9 (0.306), and YOLO-CXR (0.338). Similarly, the proposed model achieved an *mAP*@[0.5 : 0.95] of 0.196, outperforming all competing methods. In terms of detection sensitivity, our approach achieved the highest recall of 0.451, indicating that the proposed framework detected a larger proportion of true lesions compared with the other methods. A detailed per-category analysis further demonstrated the advantages of the proposed method. The proposed model achieved notable improvements in several clinically important lesion categories, including Cardiomegaly (0.926), Pleural effusion (0.447), Infiltration (0.395), Consolidation (0.387), and Nodule/Mass (0.348). In particular, the detection performance for Pulmonary fibrosis increased to 0.309, which was substantially higher than the competing methods. These improvements suggest that the proposed framework is effective in capturing complex pathological patterns in chest radiographs. Furthermore, the proposed method demonstrated strong performance in detecting small-scale lesions, which are commonly observed in chest radiographs and are often difficult to identify due to their limited spatial extent and subtle appearance. In our experiments, the proposed model achieved clear improvements in several categories that typically correspond to small or localized abnormalities, including Calcification (0.205), Nodule/Mass (0.348), and Pulmonary fibrosis (0.309). Compared with YOLO-CXR, which achieved 0.114, 0.208, and 0.095 on these categories respectively, the proposed method produced substantial gains in detection accuracy.

Small lesion detection remains a challenging task in chest X-ray analysis because small abnormalities occupy only a limited number of pixels and may be easily suppressed during deep feature extraction and down-sampling processes. In our study, the improved detection performance for these lesion types suggested that the proposed feature enhancement and adaptive feature aggregation mechanisms effectively preserved fine-grained spatial information across multiple feature scales.

#### Comparison on ChestX-ray8 dataset

To evaluate the generalization capability of our model, experiments were conducted on the ChestX-ray8 dataset. The results summarized in Table 4 indicate that our method consistently outperforms all baseline models across the evaluated metrics. Specifically, it achieves a *mAP*@0.5 of 0.195, representing an absolute improvement of 4.2% points over the best-performing baseline Mamba-YOLOvX with 0.153, highlighting a substantial gain in overall detection accuracy. Under the more stringent *mAP*@[0.5 : 0.95] metric, our method achieves the best performance of 0.0921, demonstrating robust performance across varying IoU thresholds. For medium and large object detection, our approach achieves $AP_M$ of 0.103 and $AP_L$ of 0.183, indicating its capability to accurately identify clinically relevant lesions. Furthermore, the method attains a recall of 0.247, surpassing all baselines and underscoring its ability to capture chest abnormalities. Collectively, these results demonstrate the effectiveness and strong generalization capability of the proposed method in chest X-ray object detection, particularly in medium-to-large lesion detection and overall recall performance.

**Table 4:** Performance comparison with state-of-the-art methods on the ChestX-ray8 dataset.

Model	*mAP*@0.5	*mAP*@[0.5 : 0.95]	$AP_S$	$AP_M$	$AP_L$	Recall
ACmix^45^	0.104	0.053	–	0.008	0.061	0.142
ParNet^46^	0.108	0.061	–	0.001	0.073	0.146
CoT^47^	0.133	0.062	–	0.016	0.075	0.160
DualAttNet^42^	0.145	0.071	–	0.026	0.076	0.161
Mamba-YOLOvX^48^	0.153	0.086	–	0.037	0.086	0.169
Ours	0.195	0.0921	–	0.103	0.183	0.247

**Table 5:** Comparison of average precision with state-of-the-art methods across 8 disease categories on the ChestX-ray8 dataset.

Disease labels	DualAttNet^42^	Mamba-YOLOvX^48^	Ours
Infiltrate	0.0126	0.00217	0.00175
Atelectasis	0.761	0.78	0.711
Pneumonia	0.0855	0.0103	**0.129**
Cardiomegaly	0.074	0.053	**0.21**
Effusion	0.11	0.153	**0.179**
Pneumothorax	0.0315	0.142	**0.187**
Mass	0.0717	0.0922	0.0807
Nodule	0.00524	0.00222	**0.0497**
*mAP*@0.5	0.145	0.153	**0.195**
*mAP*@[0.5 : 0.95]	0.071	0.086	**0.0921**
Recall	0.161	0.169	**0.247**

To further evaluate the performance of the model on specific disease categories, we compared DualAttNet, Mamba-YOLOvX, and our proposed method on the ChestX-ray8 dataset, as shown in Table 5. The results demonstrate that our method achieves superior performance across multiple disease types. For instance, in detecting Pneumonia, Cardiomegaly, Effusion, and Pneumothorax, the corresponding AP values are 0.129, 0.210, 0.179, and 0.187, respectively, all exceeding the baseline models and indicating high sensitivity to clinically relevant lesions. Additionally, our method shows a notable improvement in detecting Nodules, with an AP of 0.0497, reflecting its potential capability in identifying smaller abnormalities. Overall, the method achieves 0.195 in *mAP*@0.5 and 0.0921 in *mAP*@[0.5 : 0.95], with a recall of 0.247, outperforming both DualAttNet and Mamba-YOLOvX. These results indicate that the proposed approach not only improves individual disease detection but also demonstrates strong generalization and robustness in comprehensive chest abnormality detection.

These results indicate that integrating contextual information with adaptive feature focusing enhances the model’s sensitivity to subtle pathological patterns. Consequently, the proposed framework may provide more reliable detection of early-stage or small-scale abnormalities in chest radiographs.

In summary, the proposed method achieves a favorable balance between detection accuracy, scale robustness, recall capability, and model complexity on the VinDr-CXR and ChestX-ray8 datasets. Its sustained advantages in detecting small lesions and achieving high localization accuracy at elevated IoU thresholds validate the effectiveness and advanced nature of the proposed approach for multi-scale lesion detection in chest imaging tasks.

#### Error analysis and clinical relevance

**Figure 8:**
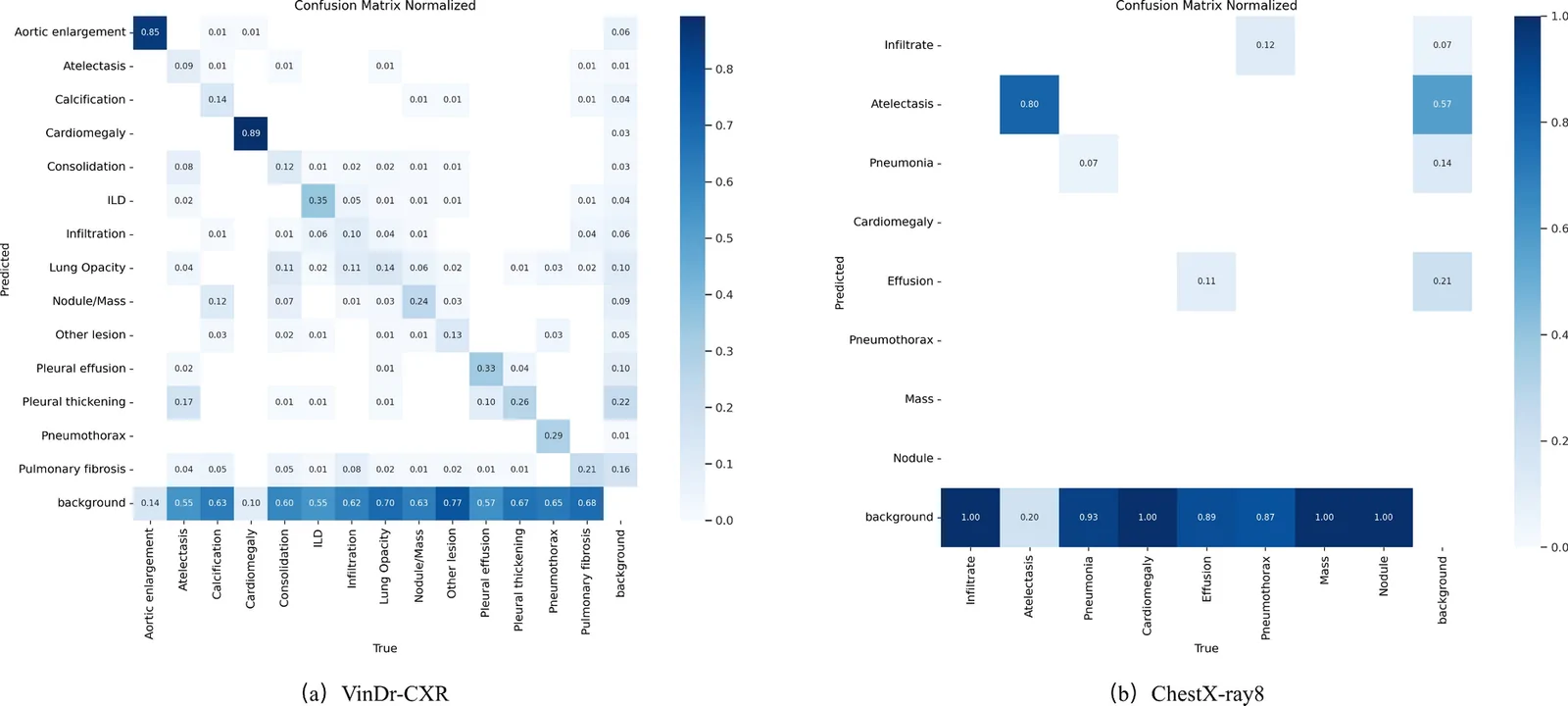
Normalized confusion matrices on the (**a**) VinDr-CXR and (**b**) ChestX-ray8 datasets. Columns denote ground-truth categories and rows denote predicted categories. The background row reflects missed detections, while the background column reflects false-positive predictions.

To further evaluate the clinical applicability of the proposed method beyond standard detection metrics, we performed a category-level error analysis using normalized confusion matrices on the VinDr-CXR and ChestX-ray8 datasets, as shown in Fig.  8. Different from only reporting overall mAP and recall, the confusion matrices provide a more detailed view of false-negative and false-positive distributions across lesion categories, allowing us to examine whether the proposed model can maintain reliable detection performance for clinically meaningful abnormalities.

As shown in Fig. 8(a), on the VinDr-CXR dataset, the proposed method achieves strong recognition performance for several visually and clinically important categories, such as Aortic enlargement and Cardiomegaly. This indicates that the proposed context-embedded feature enhancement strategy is effective in capturing global anatomical structures and distinguishing large-scale pathological patterns. Meanwhile, the model also shows meaningful responses for several challenging lesion categories with irregular morphology or subtle appearance, including Pleural effusion, Pneumothorax, Nodule/Mass, Pulmonary fibrosis, and ILD. These results suggest that the combination of adaptive multi-scale feature fusion and shape-aware optimization improves the model’s ability to detect lesions with diverse scales and shapes.

The confusion matrix also reveals that the remaining false negatives are mainly associated with categories characterized by small size, weak contrast, diffuse appearance, or ambiguous boundaries. Typical examples include Nodule/Mass, Lung Opacity, Consolidation, Infiltration, and Pulmonary fibrosis. These categories are inherently difficult because their visual patterns can be partially suppressed during feature down-sampling or confused with overlapping anatomical structures. The proposed AFFN and SAOS are specifically designed to alleviate these problems by enhancing multi-scale feature representation and improving localization stability. Therefore, the category-level error distribution further supports the motivation of the proposed method.

For the ChestX-ray8 dataset, as shown in Fig. 8(b), the confusion matrix shows that the proposed model can still identify representative abnormal categories such as Atelectasis, Effusion, Pneumonia, and Infiltrate under a more challenging annotation setting. Compared with VinDr-CXR, ChestX-ray8 contains weaker localization annotations and more ambiguous lesion boundaries, which increases the difficulty of accurate lesion localization. Nevertheless, the model maintains discriminative responses for several abnormal categories, indicating that the proposed framework has a certain degree of cross-dataset robustness.

From a clinical perspective, the analysis shows that some missed detections still occur in clinically important categories such as Pneumothorax, Nodule/Mass, Consolidation, and Pleural effusion. However, the proposed method improves the overall recall and category-level recognition ability compared with the baseline, which is important for reducing missed abnormalities in computer-aided diagnosis.

### Ablation study

To evaluate the effectiveness of the proposed components, ablation experiments were conducted on the VinDr-CXR and ChestX-ray8 datasets under the same unified experimental protocol. The baseline detector was used as the reference model, and the Context-Embedded Feature Enhancement Network (CEFEN), Adaptive Feature Focusing Network (AFFN), and Shape-Aware Optimization Strategy (SAOS) were progressively introduced.

**Table 6:** Performance comparison of module ablation experiments on the VinDr-CXR and ChestX-ray8 datasets.

Dataset	Model	CEFEN	AFFN	SAOS	*mAP*@0.5	*mAP*@[0.5 : 0.95]
VinDr-CXR	Baseline				0.327	0.154
	Baseline+	*√*			0.379	0.181
	Baseline+		*√*		0.383	0.177
	Baseline+			*√*	0.387	0.187
	Ours	*√*	*√*	*√*	**0.401**	**0.196**
ChestX-ray8	Baseline				0.144	0.0861
	Baseline+	*√*			0.176	0.0892
	Baseline+		*√*		0.187	0.0914
	Baseline+			*√*	0.189	0.0918
	Ours	*√*	*√*	*√*	**0.195**	**0.0921**

As shown in Table  6, each proposed module contributes positively to the detection performance compared with the baseline model. On the VinDr-CXR dataset, the baseline achieves 0.327 *mAP*@0.5 and 0.154 *mAP*@[0.5 : 0.95]. After introducing CEFEN, the performance improves to 0.379 and 0.181, respectively, indicating that context-embedded feature enhancement is effective in improving lesion representation under complex thoracic backgrounds. When AFFN is added individually, the model achieves 0.383 *mAP*@0.5 and 0.177 *mAP*@[0.5 : 0.95], demonstrating the benefit of adaptive multi-scale feature fusion. SAOS also improves the baseline to 0.387 *mAP*@0.5 and 0.187 *mAP*@[0.5 : 0.95], suggesting that shape-aware localization constraints are beneficial for bounding-box regression. By integrating CEFEN, AFFN, and SAOS, the full model obtains the best performance on VinDr-CXR, reaching 0.401 *mAP*@0.5 and 0.196 *mAP*@[0.5 : 0.95].

A similar trend can be observed on the ChestX-ray8 dataset. Compared with the baseline, all three individual modules improve *mAP*@0.5. Specifically, CEFEN, AFFN, and SAOS improve *mAP*@0.5 from 0.144 to 0.176, 0.187, and 0.189, respectively. The full model achieves the highest *mAP*@0.5 of 0.195, indicating improved detection capability under the ChestX-ray8 setting. For the stricter *mAP*@[0.5 : 0.95] metric, AFFN and SAOS achieve 0.0892 and 0.0914, respectively, while the full model obtains 0.0921. This result suggests that the stricter localization metric on ChestX-ray8 is more sensitive to dataset annotation characteristics and module interactions. Nevertheless, the overall ablation results demonstrate that the proposed components consistently enhance the baseline detector, especially in terms of *mAP*@0.5.

To evaluate the effectiveness of the proposed modules and further investigate the contribution of their internal design choices, ablation experiments were conducted on the VinDr-CXR and ChestX-ray8 datasets under the same unified experimental protocol. The baseline detector was used as the reference model. We first evaluated the complete versions of the three proposed modules, namely the Context-Embedded Feature Enhancement Network (CEFEN), the Adaptive Feature Focusing Network (AFFN), and the Shape-Aware Optimization Strategy (SAOS). Then, fine-grained ablation experiments were performed to isolate several key internal components, including the global-context branch in CEFEN, CBAM and ASF in AFFN, and NWD and shape-weighted constraints in SAOS. The results are summarized in Table  7. Here, “w/o” denotes “without”.

**Table 7:** Fine-grained module ablation results on the VinDr-CXR and ChestX-ray8 datasets.

Dataset	Model	Ablation setting	*mAP*@0.5	*mAP*@[0.5 : 0.95]
VinDr-CXR	Baseline	Original baseline detector	0.327	0.154
	Baseline+CEFEN	w/o global-context branch	0.361	0.172
	Baseline+CEFEN	Complete CEFEN	0.379	0.181
	Baseline+AFFN	w/o CBAM	0.374	0.176
	Baseline+AFFN	w/o ASF	0.365	0.174
	Baseline+AFFN	Complete AFFN	0.383	0.177
	Baseline+SAOS	w/o NWD	0.369	0.178
	Baseline+SAOS	w/o shape weights	0.377	0.180
	Baseline+SAOS	Complete SAOS	0.387	0.187
	Full model	CEFEN + AFFN + SAOS	**0.401**	**0.196**
ChestX-ray8	Baseline	Original baseline detector	0.144	0.0861
	Baseline+CEFEN	w/o global-context branch	0.164	0.0877
	Baseline+CEFEN	Complete CEFEN	0.176	0.0892
	Baseline+AFFN	w/o CBAM	0.180	0.0904
	Baseline+AFFN	w/o ASF	0.171	0.0891
	Baseline+AFFN	Complete AFFN	0.187	0.0914
	Baseline+SAOS	w/o NWD	0.172	0.0895
	Baseline+SAOS	w/o shape weights	0.181	0.0906
	Baseline+SAOS	Complete SAOS	0.189	0.0918
	Full model	CEFEN + AFFN + SAOS	**0.195**	**0.0921**

On VinDr-CXR, the baseline achieves 0.327 *mAP*@0.5 and 0.154 *mAP*@[0.5 : 0.95]. After introducing the complete CEFEN, the performance increases to 0.379 and 0.181, respectively. When the global-context branch is removed, the performance decreases to 0.361 and 0.172, confirming that global anatomical context is beneficial for improving lesion representation in complex thoracic backgrounds.

For AFFN, the complete module achieves 0.383 *mAP*@0.5 on VinDr-CXR. Removing CBAM reduces the performance to 0.374, while removing ASF further decreases it to 0.365. This indicates that both CBAM and ASF contribute to multi-scale feature enhancement, with ASF playing a more prominent role in adaptive feature aggregation. A similar trend can be observed on ChestX-ray8, where AFFN without ASF obtains 0.171 *mAP*@0.5, which is lower than AFFN without CBAM. These results suggest that adaptive spatial fusion is important for improving feature alignment across different lesion scales.

For SAOS, both NWD and shape-weighted constraints contribute to localization performance. On VinDr-CXR, removing NWD reduces *mAP*@0.5 to 0.369, while removing shape weights results in 0.377. The complete SAOS achieves 0.387 *mAP*@0.5 and 0.187 *mAP*@[0.5 : 0.95], demonstrating that spatial distribution modeling and shape-aware constraints are complementary for bounding-box regression. On ChestX-ray8, the complete SAOS also achieves the strongest single-module performance, reaching 0.189 *mAP*@0.5 and 0.0918 *mAP*@[0.5 : 0.95].

**Figure 9:**
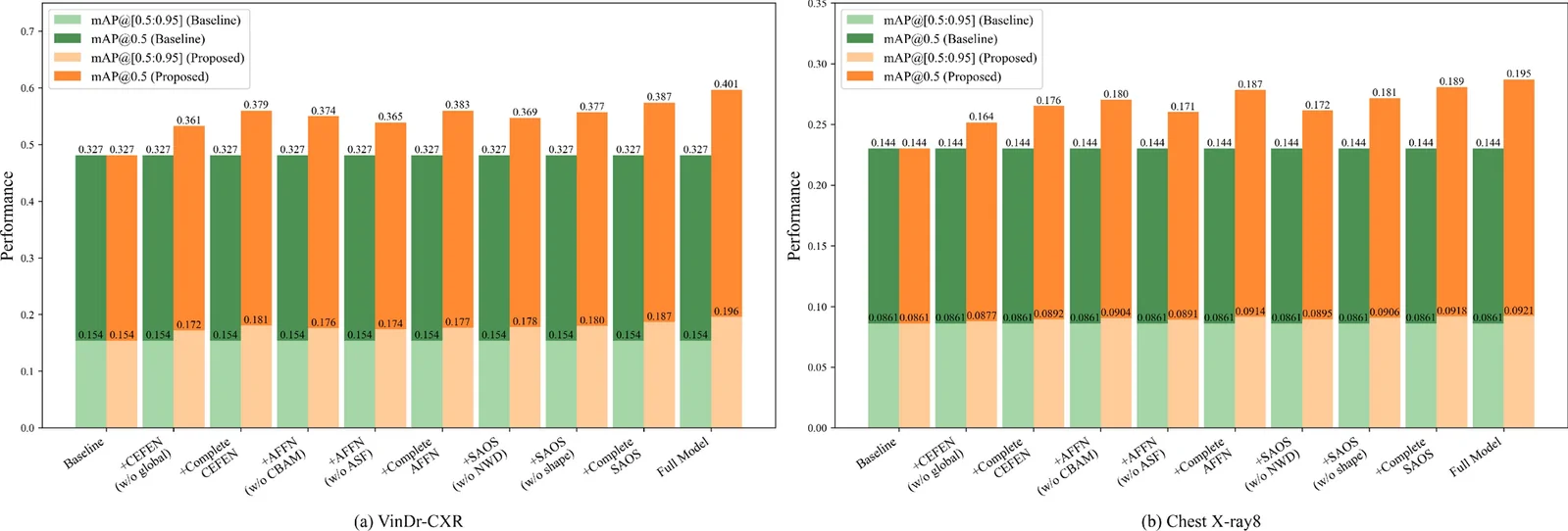
Visual comparison of the fine-grained ablation results on the (a) VinDr-CXR and (b) ChestX-ray8 datasets. The figure shows the performance variations of different ablation settings in terms of *mAP*@0.5 and *mAP*@[0.5 : 0.95], compared with the baseline model.

To provide a more intuitive illustration of the fine-grained ablation results, the performance variations of different ablation settings are visualized in Figure 9. The figure compares the baseline and the corresponding ablation variants in terms of *mAP*@0.5 and *mAP*@[0.5 : 0.95] on the VinDr-CXR and ChestX-ray8 datasets. As shown in Figure 9, the complete versions of CEFEN, AFFN, and SAOS generally outperform their corresponding incomplete variants.

When all three modules are integrated, the full model achieves the best *mAP*@0.5 on both datasets, reaching 0.401 on VinDr-CXR and 0.195 on ChestX-ray8. On VinDr-CXR, the full model also obtains the highest *mAP*@[0.5 : 0.95] of 0.196, indicating improved detection accuracy and localization robustness. On ChestX-ray8, the full model also achieves the highest *mAP*@0.5 and *mAP*@[0.5 : 0.95], reaching 0.195 and 0.0921, respectively. Although the improvement in *mAP*@[0.5 : 0.95] is relatively modest, it still indicates that the integration of CEFEN, AFFN, and SAOS improves overall detection and localization performance. This suggests that the stricter localization metric may be more sensitive to dataset annotation characteristics and the interaction among different modules. Nevertheless, the overall results demonstrate that CEFEN, AFFN, and SAOS are effective components for improving chest lesion detection, and the fine-grained ablation further confirms the necessity of the global-context branch, ASF, NWD, and shape-weighted constraints.

**Figure 10:**
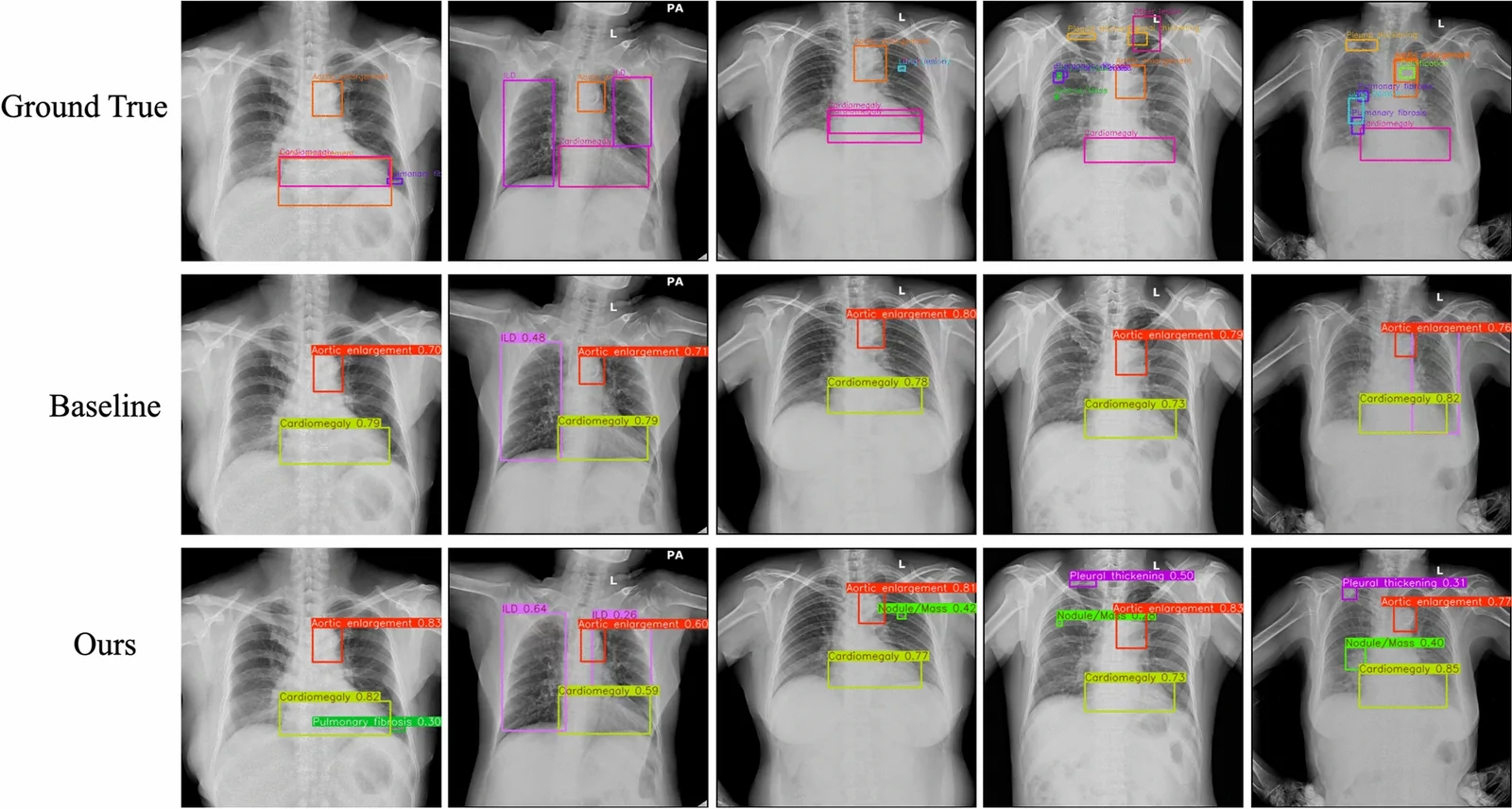
Qualitative detection results on the VinDr-CXR test set. The rows show ground-truth annotations, baseline predictions, and predictions of the proposed method. The proposed method produces more accurate bounding boxes for small, low-contrast, and irregular lesions.

To visually demonstrate the detection performance of the proposed model on real chest images, Figure 10 presents representative visual detection results from the VinDr-CXR test set. The figure compares the bounding boxes predicted by the model with the ground-truth bounding boxes annotated by radiologists, covering lesions from different disease categories, scales, and morphological characteristics.

When all three modules are integrated, the full model achieves the best performance on both datasets. Specifically, it reaches 0.401 *mAP*@0.5 and 0.196 *mAP*@[0.5 : 0.95] on VinDr-CXR, and 0.195 *mAP*@0.5 and 0.0921 *mAP*@[0.5 : 0.95] on ChestX-ray8. These results demonstrate that CEFEN, AFFN, and SAOS are effective and complementary components for improving chest lesion detection. The fine-grained ablation results further confirm the necessity of the global-context branch, ASF, NWD, and shape-weighted constraints.

## Discussion

The proposed adaptive collaborative feature fusion and shape-aware optimization framework achieved consistent improvements over state-of-the-art chest lesion detection methods on both the VinDr-CXR and ChestX-ray8 datasets. These results demonstrate that the proposed method is effective not only on a high-quality radiologist-annotated dataset, but also under different data distributions and annotation characteristics. The improvements in mAP, localization accuracy, and recall indicate that the proposed framework can enhance lesion recognition while reducing missed detections, which is particularly important for multi-scale chest lesion detection.

Chest X-ray lesion detection remains challenging because lesions often exhibit large variations in scale, irregular morphology, weak contrast, and complex spatial relationships with surrounding anatomical structures. Conventional detectors usually rely on fixed feature fusion strategies or overlap-based localization objectives, which may limit their sensitivity to small, diffuse, or irregular lesions. In contrast, the proposed framework explicitly addresses these challenges through three complementary mechanisms. CEFEN integrates global anatomical context with local lesion details to improve feature discrimination in complex thoracic backgrounds. AFFN performs adaptive spatial aggregation across multi-scale feature maps, enhancing cross-scale feature interaction and improving the representation of lesions with different sizes and locations. SAOS combines normalized Wasserstein distance with shape-weighted constraints, which stabilizes bounding-box regression and improves localization robustness for irregular lesions.

The ablation studies further confirm the effectiveness and complementarity of the proposed modules. The performance gains obtained by progressively introducing CEFEN, AFFN, and SAOS show that feature enhancement, adaptive multi-scale fusion, and shape-aware localization each contribute to the final detection performance. The fine-grained ablation results also verify the importance of the global-context branch, adaptive spatial fusion, normalized Wasserstein distance, and shape-weighted constraints. These findings suggest that jointly optimizing contextual representation, scale adaptability, and geometric localization is more effective than relying only on deeper backbones or static feature pyramid structures.

Although the proposed method achieves promising results, several challenging cases remain, especially extremely small, low-contrast, or anatomically overlapping lesions. Future work will further explore refined small-lesion feature enhancement, anatomical prior modeling, confidence calibration, and uncertainty estimation to improve the reliability and clinical applicability of the proposed framework.

## Conclusions

This study proposed an adaptive collaborative feature fusion and shape-aware optimization framework for multi-scale chest lesion detection in chest X-ray images. The proposed method aims to address several key challenges in thoracic disease detection, including large lesion-scale variations, complex anatomical backgrounds, missed detection of small lesions, and localization instability for irregular lesions.

The framework integrates three complementary components: the Context-Embedded Feature Enhancement Network (CEFEN), the Adaptive Feature Focusing Network (AFFN), and the Shape-Aware Optimization Strategy (SAOS). CEFEN enhances lesion representation by jointly modeling global anatomical context and local lesion details. AFFN improves multi-scale feature aggregation through adaptive spatial fusion, enabling the model to better handle lesions with different sizes and locations. SAOS introduces shape-aware localization optimization by combining normalized Wasserstein distance with shape-weighted constraints, thereby improving bounding-box regression stability for small and irregular lesions.

Experimental results on the VinDr-CXR and ChestX-ray8 datasets demonstrate the effectiveness and generalization capability of the proposed method. Compared with state-of-the-art methods, the proposed framework achieves improved detection accuracy, recall performance, and localization robustness across different lesion scales. The ablation studies further confirm the contribution and complementarity of CEFEN, AFFN, and SAOS.

Overall, the proposed framework provides an effective solution for multi-scale chest lesion detection in radiographic images. These results indicate that jointly optimizing contextual feature representation, adaptive multi-scale fusion, and shape-aware localization is beneficial for improving medical object detection systems. Future work will further explore refined small-lesion feature enhancement, anatomical prior modeling, confidence calibration, uncertainty estimation, and computational efficiency optimization to improve the reliability and clinical applicability of the proposed framework.

## Data Availability

The datasets utilized in the present investigation are publicly accessible and can be found at https://www.physionet.org/content/vindr-cxr/1.0.0/ and https://nihcc.app.box.com/v/ChestXray-NIHCC
